# A Review on the Mechanical Modeling of Composite Manufacturing Processes

**DOI:** 10.1007/s11831-016-9167-2

**Published:** 2016-01-20

**Authors:** Ismet Baran, Kenan Cinar, Nuri Ersoy, Remko Akkerman, Jesper H. Hattel

**Affiliations:** 1 Faculty of Engineering, University of Twente, 7500 AE Enschede, The Netherlands; 2 Mechanical Engineering Department, Namik Kemal University, 59100 Tekirdag, Turkey; 3 Mechanical Engineering Department, Bogazici University, 34342 Istanbul, Turkey; 4 Department of Mechanical Engineering, Technical University of Denmark, 2800 Lyngby, Denmark

## Abstract

The increased usage of fiber reinforced polymer composites in load bearing applications requires a detailed understanding of the process induced residual stresses and their effect on the shape distortions. This is utmost necessary in order to have more reliable composite manufacturing since the residual stresses alter the internal stress level of the composite part during the service life and the residual shape distortions may lead to not meeting the desired geometrical tolerances. The occurrence of residual stresses during the manufacturing process inherently contains diverse interactions between the involved physical phenomena mainly related to material flow, heat transfer and polymerization or crystallization. Development of numerical process models is required for virtual design and optimization of the composite manufacturing process which avoids the expensive trial-and-error based approaches. The process models as well as applications focusing on the prediction of residual stresses and shape distortions taking place in composite manufacturing are discussed in this study. The applications on both thermoset and thermoplastic based composites are reviewed in detail.

## Introduction

Fiber reinforced composite materials have been increasingly used in various structural components in the aerospace, marine, automotive and wind energy sectors. Although manufacturing and investment costs of composite materials are high when compared to conventional materials (primarily metals), their higher strength per unit weight and fewer required machining and fastening operations increase the popularity of composite materials day by day. The direction-dependent mechanical properties of composite materials can also be advantageous in some applications where strength is only required in a specific direction.

In the processing of composite materials, the final shape of the composite parts is not the same as the mould shape after the process due to process induced distortions. The basic reason behind the distortion is the process induced residual stresses occurring during the manufacturing process. The nonuniform distribution of residual stresses inside the composite materials results in deformation, matrix cracking, and even delamination. These distortions are represented by spring-in in curved parts and by warpage in flat parts. Problems occur during and after the assembly of parts due to poor contact between mating surfaces unless the magnitude of these distortions are predicted within the tolerances. The assembly of aero-structures especially rigid structures requires matching of smaller sub-components like shims in the assembly phase. Using the sub-components causes the assembly of composite structures to remain a labor-intensive task. On the manufacturing floor, a trial and error approach is preferred to compensate geometrical variations like spring-in angle, but this method is very expensive and time consuming when manufacturing of large components. If the distortions are predicted closely in advance, the investment to the trial and error modification and labor-intensive task during assembly phase can be prevented.

The most common problem is that geometrical variations may depend on lay-up, material, processing temperature, tooling geometry etc., which makes the problem more complex. To solve this problem requires more powerful computational models. Increasing the simulation capacity for the manufacturing processes is an important step towards a more cost efficient development and manufacturing of composite structures.

Some attractive examples from industry can be given, where the above mentioned problems occur during manufacturing. The 27 m long A350 XWB rear wing spar forms the structural heart of the aircraft wing’s fixed trailing edge,holding vital parts such as the main landing gear. Aileron ribs and fuselage stringers also can be given as examples in the same context. In aerospace applications, the autoclave manufacturing method is used to manufacture these structures. Similar structures can be manufactured using the pultrusion process if the cross section of the structure is constant. Vertical axis wind turbine blades can be an innovative example [1, 2]. Manufacturing large blades in one step using a single die will lead to cost reduction for large series production. In addition to these manufacturing methods, the resin transfer moulding (RTM) method can also be used for manufacturing of aileron rib. The inboard flaperon of the BA609 (Bell Agusta), which is an aerodynamic control surface also presents different critical features. In fact, the inboard flaperon is a primary component so the reliability must be nearly absolute [3].

Developing computational models requires deep knowledge of the mechanisms generating the geometrical variations. In this context residual stresses developed during manufacturing should be examined. Residual stresses can be categorized according to the scale at which they originate and whether they are thermoelastic or non-thermoelastic. Residual stresses can be grouped as micro scale residual stresses and macro scale residual stresses. Micro scale residual stresses develop between the fibers and resin as a consequence of (*i*) thermal expansion mismatch between the fibers and resin, (*ii*) chemical shrinkage of the resin during polymerization, and (*iii*) moisture absorption. The residual stresses at this scale do not cause any distortions of the composite laminate although they adversely affect the strength of the laminate by matrix cracking. The stresses at this scale are self-equilibrating so that they do not lead to large deformations. On the other hand, residual stresses at the macro scale are the source of large dimensional changes. Anisotropic behaviour of individual plies, the constraint effect of individual plies, and tooling constraints are the main sources that trigger the residual stresses at this scale.

Thermoelastic residual stresses are reversible so that the distortion can be eliminated by heating the part to its polymerizarion temperature. The source of these stresses in composite materials is the difference between in-plane thermal strains and through-thickness thermal strains. Non-thermoelastic residual stresses, on the other side, are irreversible and the mechanisms behind them are more complex. These mechanisms can be listed as follows [4–8]: (*i*) the tool–part interaction, (*ii*) chemical shrinkage during polymerization, (*iii*) consolidation, (*iv*) through-thickness degree of cure or crystallinity gradients, and (*v*) fiber volume fraction gradients.

In the present study, the main mechanisms generating the residual stresses and shape distortions are explained in detail. The state-of-the-art computational approaches are reviewed for modelling the constitutive behaviour as well as the general multiphysics phenomena governing composite manufacturing processes. The interaction between the composite part and the tool is also explained. This work also provides a general overview of the applications of the mechanical process modelling in fiber reinforced thermoset as well as thermoplastic composites. Since the primary focus of this review paper is on the process models to predict the residual stresses and shape distortions during composites manufacturing, phenomena such as intimate contact, bonding, void growth, and polymer degradation and the related models are not reviewed here.

## Mechanisms Generating Residual Stresses and Geometrical Variations

Process induced residual stresses and deformations are inevitable during the processing of composite materials and there are several studies carried out in the literature on this particular subject. These studies can be grouped into two basic categories: studies on clarifying the mechanisms behind process induced residual stresses and deformations, and studies on predicting these deformations through different numerical and analytical methods.

**Fig. 1:**
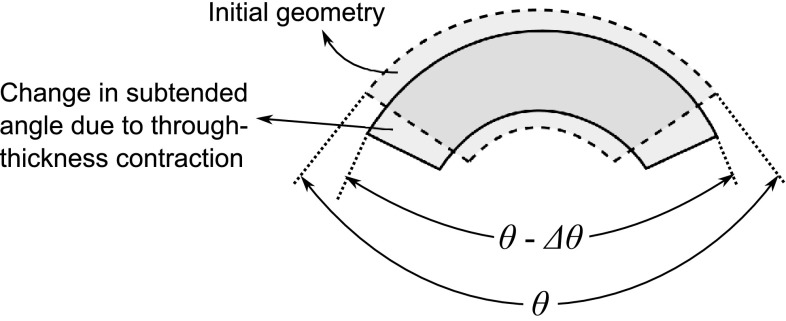
A reduction in enclosed angle $\theta$ due to contraction resulting in the well-known spring-in deformation pattern

**Fig. 2:**
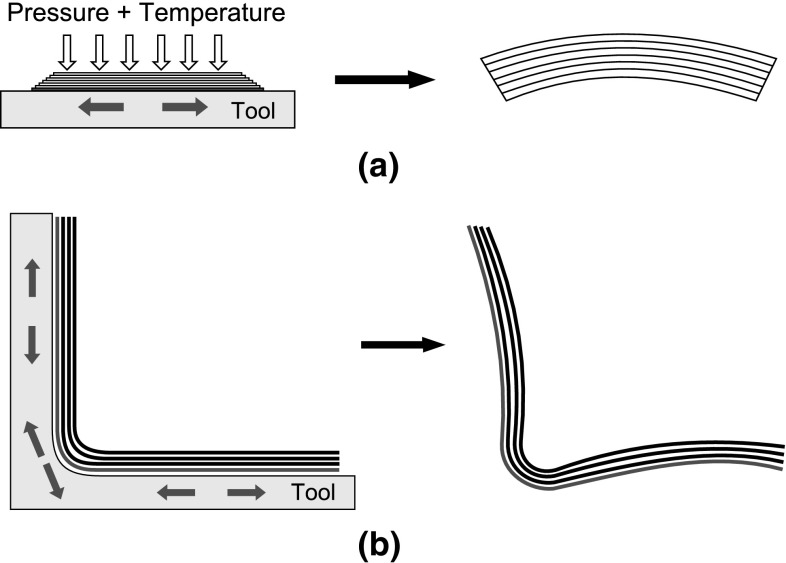
Effect of tool–part interaction on distortion. **a** Flat parts. **b** Parts that have corner sections

As mentioned before, it is necessary to have a better understanding of the process induced residual stresses since they directly affect the residual shape deformations which are critical for dimensional tolerances. There are various mechanisms that are responsible for the development of residual stresses and distortions. Thermal anisotropy, chemical shrinkage of the resin, tool–part interaction, resin flow, consolidation and compaction, fiber volume fraction gradients, moisture swelling, prepreg variability, gradients in temperature and the degree of cure or crystallization have all been identified as mechanisms responsible for process induced residual stresses. In the following sections, these mechanisms are discussed in more detail together with corresponding references from literature.

### Thermal Anisotropy

The difference between the coefficient of thermal expansion (CTE) of the fiber and resin causes residual stresses on both micro and macro scale; however, this does not cause any distortion on the macro scale [4]. The CTE of the fibers is much smaller than that of the resin. This mismatch is resulting in the thermal anisotropy on the macro scale because the part expands or contracts more in the resin dominated direction as compared to the fiber dominated direction. A balanced symmetric flat part does not have any out of plane distortion if there is no tooling constraint. However, the CTE difference between the thickness direction and the circumferential direction results in a reduction in the enclosed angle of the part for curved regions which is known as spring-in. In the case of isotropic materials, the contraction upon cooling in a curved region is uniform, and therefore the angle is preserved. The CTE and strains in the through-thickness direction are much higher than the CTE and stains in the fiber direction for fiber reinforced composites. To illustrate, this leads to a reduction in the enclosed angle ($\theta$) as shown in Fig. 1 in which the effect is reversible, i.e., the spring-in angle reduces if the part is reheated.

The first attempt to calculate the magnitude of the enclosed angle was proposed by Nelson and Cairns [9] with the following Eq. 1:

1 $$\begin{aligned} \Delta \theta =\theta \frac{(\alpha _\theta -\alpha _R)\Delta T}{1+\alpha _R \Delta T} \end{aligned}$$

where $\Delta \theta$ is the spring-in angle, $\theta$ is the subtended angle of the part, $\alpha _\theta$ is the circumferential coefficient of thermal expansion, $\alpha _R$ is the radial coefficient of thermal expansion, and $\Delta T$ is the temperature change.

The development of residual stresses and distortions in unsymmetrical flat [10, 11] and symmetric curved laminates [12, 13] was monitored by interrupting the cure cycle at pre-determined points. By this method, the thermoelastic and non-thermoelastic components of the spring-in during curing can be determined. The stress free temperature of the composite samples was measured in [11] and it was found that the stress free temperature of samples cured beyond vitrification was higher than their cure temperature, which showed that a certain percentage of non-thermoelastic stress was present in the composite part. It was concluded that out of plane deformations of the flat composite laminates were small when the laminates were cured at a low temperature [10]. The deformation increased very sharply during the second heating ramp of the manufacturer recommended cure cycle (MRCC) [11]. It was also found in [11] that the transverse CTE remained almost constant below the glass transition temperature. Ersoy et al. [13] adopted a cure quench technique to analyze the development of spring-in angle during the curing of an AS4/8552 thermosetting composite. In their experiments, C-shaped laminates were cured on the inner wall of an aluminum tube. It was found that the specimens quenched before vitrification had a larger spring-in angle than the samples quenched after vitrification. According to their explanation, in the rubbery state (above the glass transition temperature) the CTE of the composite part was larger than the CTE in the glassy state. Therefore, quenching the samples in the rubbery state caused the samples to shrink more, and in turn, to spring-in more. It was also observed in [13] that the thermoelastic component of the spring-in was 50 % of the final spring-in with the remaining non-thermoelastic component being mainly due to cure shrinkage. A similar mechanism that incorporates the higher CTE of the composite part in the rubbery state was proposed by Svanberg and Holmberg [12] to show the spring-out phenomena during post-curing of partially cured curved parts produced by the RTM. This production method is different from the prepreg layup method. In this method, the resin is injected into a mould that consists of two rigid mould halves (the female and male moulds), and the mould is then heated. They observed that an increase in the cure temperature led to more spring-in because a high cure temperature contributed to larger thermal strains and a higher degree of cure. This means that the stress level and the corresponding frozen strains at vitrification is higher for a higher in-mould cure temperature.

**Fig. 3:**
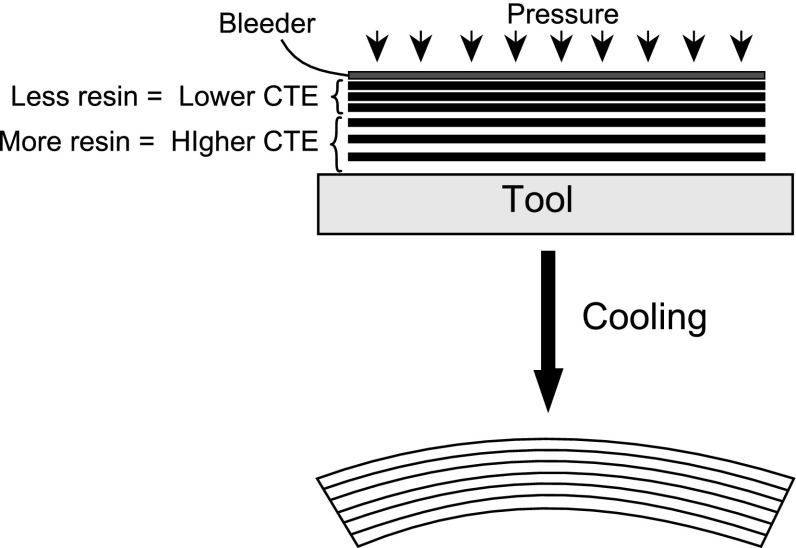
Effect of resin flow on the warpage in flat laminates

**Fig. 4:**
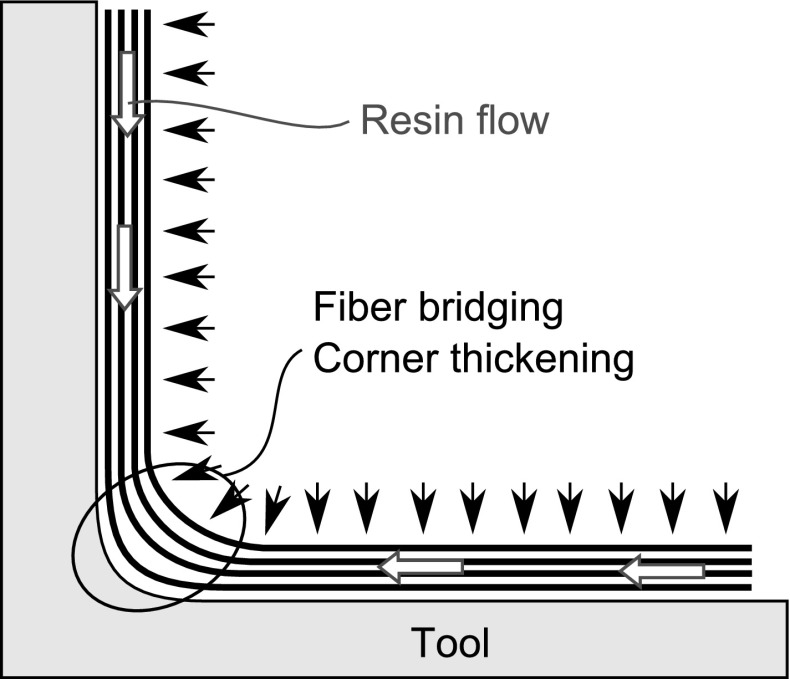
Corner thickening due to resin flow from arms to the corner

Radford and Rennick [14] proposed another method to quantify the thermoelastic and non-thermoelastic components of spring-in and to study the thermoelastic behaviour of composite angle brackets with varying laminate thicknesses, stacking sequences and part radii. A similar technique was then used by Garstka [15] as well. A laser reflection method was used to measure the spring-in angle as the sample was exposed to a temperature change in an oven. It was found that the thermoelastic effect was independent of the laminate thickness and corner radius; however, it was affected by the laminate stacking sequence. Slightly different results were found in [15] related to the effect of thickness on the thermoelastic component of the spring-in. Experimental results showed a small increase in the thermoelastic spring-in for thicker laminates due to the corner thickening caused by the resin percolation during processing.

Residual stresses due to the thermal anisotropy were modelled in [16–20] by considering the cool-down stage of the curing process and the stresses occuring during the curing were neglected. Hahn and Pagano [16] used a linear elastic constitutive model to determine the curing stresses in symmetrical boron-epoxy composite laminates. The residual stresses were calculated using the classical laminated theory (CLT) [16]. It was assumed that the part was stress free up to the highest curing temperature prior to the final cool-down stage due to low stiffness of the resin. A linear viscoelastic constitutive model was used to examine the effect of viscoelastic relaxation of the resin on the development of residual stresses during the cool-down stage [17–19]. Weitsman concluded that the residual stresses were reduced by more than 20 % [17] due to viscoelastic relaxation.

### Polymerization/Crystallization Shrinkage

Crystallization causes shrinkage in thermoplastic composites whereas curing is the main cause of shrinkage in thermosetting resins before cooling. In thermosetting polymers during polymerization, the liquid resin is converted into a hard brittle solid by chemical cross-linking which increases the density and reduces the volume [21]. Resin shrinkage only occurs during the curing process and ceases once curing is complete. The amount of composite shrinkage during curing varies among the in-plane directions and the through-thickness direction due to the constraints provided by the fibers. Shrinkage strains will be much larger in the transverse direction than the strains in the fiber direction. Hence, the effect of the chemical shrinkage on residual stress and deformation is very similar to the effect of thermal contraction on residual stress and deformation, and can be analyzed in the same way. In order to take the effect of cure shrinkage on the spring-in into account, Radford and Diefendorf [22] added a cure shrinkage term into Eq. 1 and the spring-in angle is then expressed as:

2 $$\begin{aligned} \Delta \theta =\theta \left[ \frac{(\alpha _\theta -\alpha _R)\Delta T}{1+\alpha _R \Delta T} + \frac{\varepsilon _\theta -\varepsilon _R}{1+\varepsilon _R}\right] \end{aligned}$$

where $\varepsilon _\theta$ is the in-plane chemical shrinkage strain and $\varepsilon _R$ is the through-the-thickness chemical strain.

### Tool–Part Interaction

When the tool or mold and the composite part are forced together by a certain pressure and subjected to a temperature ramp, a shear interaction occurs between them due to the mismatch in their respective CTEs. As this occurs prior to any significant degree of resin modulus development, the shear modulus of the composite part is relatively low. The shear interaction takes place at the tool-part interface, hence the regions in the composite part not interacting with the tool does not experience this shear interaction. This results in a non-uniform stress distribution which is locked in as the resin cures. These stresses cause bending moments upon removal of the composite part from the tooling which leads to shape distortions such as warpage in the composite part. This is illustrated in Fig. 2 for generic flat and curved parts. The tool–part interaction which comes from the tooling constraints is an extrinsic source of residual stresses and shape deformations. Conversely, the thermal anisotropy and cure shrinkage are considered to be due to the intrinsic properties of the composite material itself.

### Resin Flow and Compaction

Among the large number of multipyhsical phenomena taking place during composite manufacturing processes, resin flow is another important aspect affecting the stress-strain generation. Resin flow affects the distribution of the fiber volume fraction, the mechanical properties of the laminate and the final dimensions of the part [23]. Stress calculations require knowledge of the local elastic properties which are functions of the local fiber volume fraction. Resin rich and resin poor regions occur as a consequence of resin flow within the part. The distributions of the resin flow and resin pressure in the composite part play a critical role for the void formation and migration. Resin flow is also crucial during the manufacturing of composite sandwich panels since the liquid resin pressure may cause surface dimpling and core buckling [23]. In order to increase the fiber volume fraction of the laminate, a bleeder is sometimes applied inside the vacuum bag during the manufacturing of composite laminates. Liquid resin allows the bleeder to move and bleed through the thickness direction of the laminate. Consequently, local fiber volume fraction gradients occur inside the laminate. To illustrate, flat composite parts often form resin rich regions near the tooling and resin poor regions on the bag side of the laminate, as represented schematically in Fig. 3. The local CTE of the composite part depends on the fiber volume fraction distributions. And hence, the low CTE on the upper side of the laminate results in less shrinkage than the CTE on the lower side of the laminate during the cool down. This unsymmetrical behaviour causes warpage of the flat parts as schematically shown in Fig. 3.

**Fig. 5:**
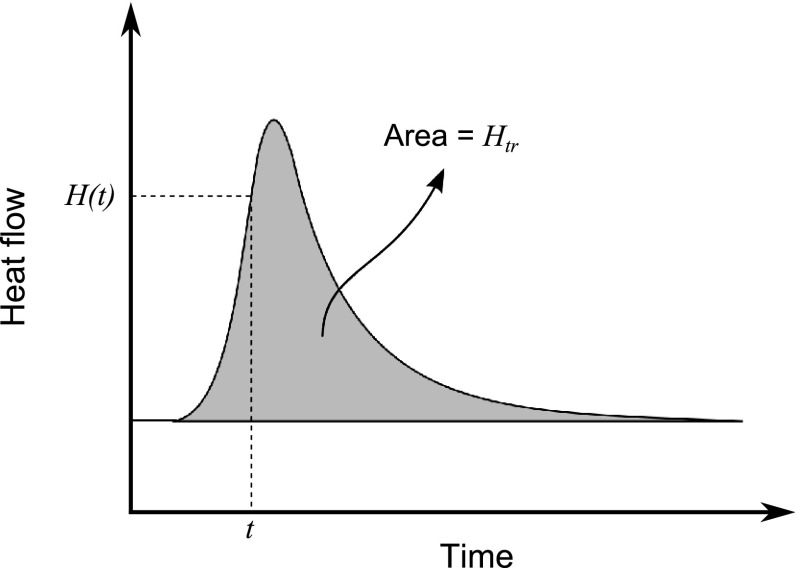
Schematic description of a DSC scan for the heat generated during curing or crystallization. *H*(*t*) is the heat flow until the time *t*, $H_{tr}$ is the total heat flow

**Fig. 6:**
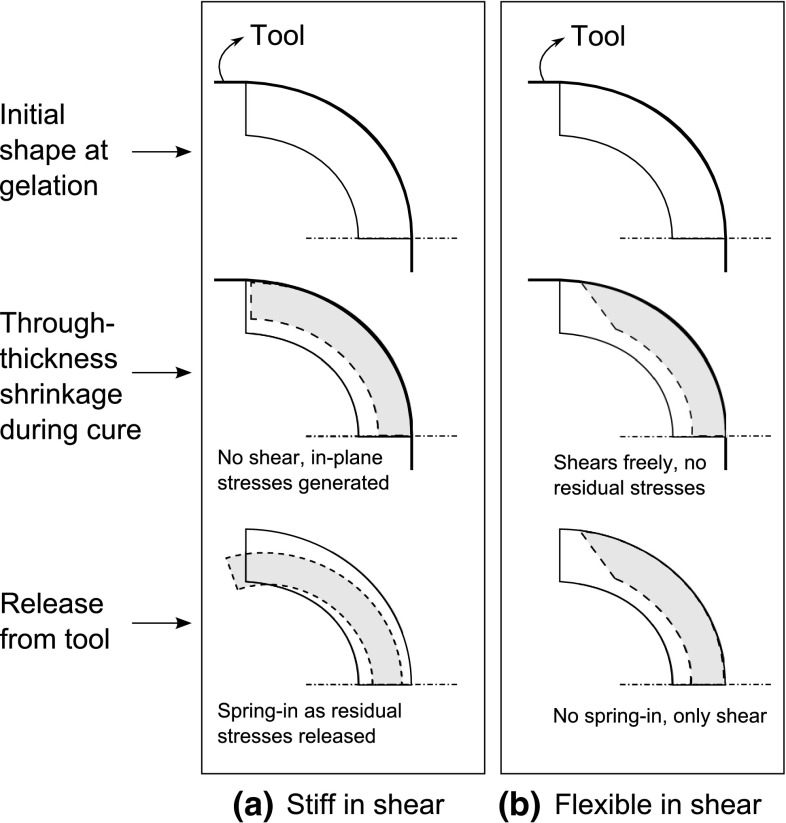
The effect of through-thickness contraction on spring-in angle. **a** Stiff in shear. **b** No restriction in shear [82]

Although the compaction mechanism for curved parts is similar to the compaction mechanism for flat parts, a different mechanism, known as fiber bridging, is responsible for the non-uniform fiber volume fraction in the through-thickness direction. As the thickness of the part is reduced by compaction, the friction between the prepreg layers prevents these layers from conforming to the tool shape at the corner. The applied pressure is ineffective at the corner of the part due to fiber bridging. This creates a low pressure region at the corner of the tool which is then percolated by resin, increasing the thickness of the part at the corner and forming a resin rich region as represented in Fig. 4. This effect is more pronounced in tighter radius parts. The corner thickening results in a higher resin fraction at the corner and hence a higher through-thickness CTE. The higher CTE at the corner in turn causes more spring-in since there is more shrinkage in the through-thickness direction during the cool down stage.

Radford [24] observed warping in symmetric carbon fiber/epoxy laminates even though classical laminate plate theory predicted no warpage. The non-uniform fiber volume fraction in the through-thickness direction, i.e., the local resin-rich regions near the tooling and resin-poor regions at the top surface adjacent to the bleeder, resulted in concave down parts. Fiber volume fractions of 0.52 and 0.59 were observed on the bag and tool sides, respectively, with an interior volume fraction of 0.57. The variation in the fiber volume fraction was included in the CLT analysis in which the mid-plane curvatures were predicted taking the CTE of the laminate and matrix shrinkage into account [24]. The predicted curvature for the long uniaxial carbon fiber/epoxy sample strips of varying thickness was found to match with the experimentally observed curvature. Furthermore, the results showed that the fiber volume fraction gradients induced during a top bleed curing was an important component of the warpage observed in the composite part [24]. Darrow and Smith [25] experimentally examined the effect of the fiber volume fraction gradient on an L-shaped laminate by applying a vacuum bag to the part with and without bleeder. Their model and experiment were compared for a unidirectional part with a 3 mm bend radius, and it was observed that the effect of the fiber volume gradient on the spring-in was smaller for thicker parts than thinner ones.

Hubert and Poursartip [23] performed an experimental investigation of the compaction of angled composite laminates using two types of material, low viscosity AS4/3501-6 and high viscosity AS4-8552. The laminates with low viscosity resin had more resin loss than those with the high viscosity resin. The total compaction strain for the low viscosity resin was caused by percolation flow under the bleed condition, while the total compaction strain for the high viscosity resin was caused by the collapse of voids. The laminate containing the low viscosity resin was analysed to determine the fiber volume fraction gradients for processes with or without using a bleeder. The data obtained from the experiments indicated that the fiber volume fraction was relatively low on the tool side and high on the bag side in the bleed condition. The fiber volume fraction measurements in the through-thickness and longitudinal directions showed that a percolation flow occurs from the tool to the bleeder. For the part manufactured in the convex tool in the no-bleed condition there was a small amount of internal percolation flow from the corner to the flat section. They also observed that the parts manufactured on the convex tool had corner thinning, whereas the parts manufactured on the concave tool had corner thickening. In the case of the convex tool, the higher reaction stress in the corner led to thinning; conversely, the concave tool had a lower reaction stress due to fiber bridging, leading to corner thickening.

### Fiber Wrinkling

The increased use of fiber reinforced composite materials in different areas encourages manufacturers to produce parts with more complex shapes. This is a difficult task due to undesired defects that often occur during manufacturing. For example, the wrinkling or buckling of plies during the lay-up of prepreg-based multilayer composites, is regularly observed in the corner sections of the L-shaped parts [26–28]. These wrinkles have a negative effect on the strength of the composite parts [29–31] and directly affect the amount of deformation after curing [32]. The elimination of wrinkles is a challenging task especially for composites parts manufactured in concave tools. Potter et al. [33] studied fiber straightness by direct measurement of fiber misalignments in prepregs and by considering the tensile load response of the uncured prepregs. The fiber misalignment in as-delivered prepreg was examined by photographic images of the surface of the uncured prepreg and by photographic images of flat laminates after curing the prepreg. The observations showed that the as-delivered prepreg material had fiber waviness which was examined also by a simple tensile test. The lead-in region from their load-displacement graph showed that there was a fiber waviness within the uncured prepreg.

**Fig. 7:**
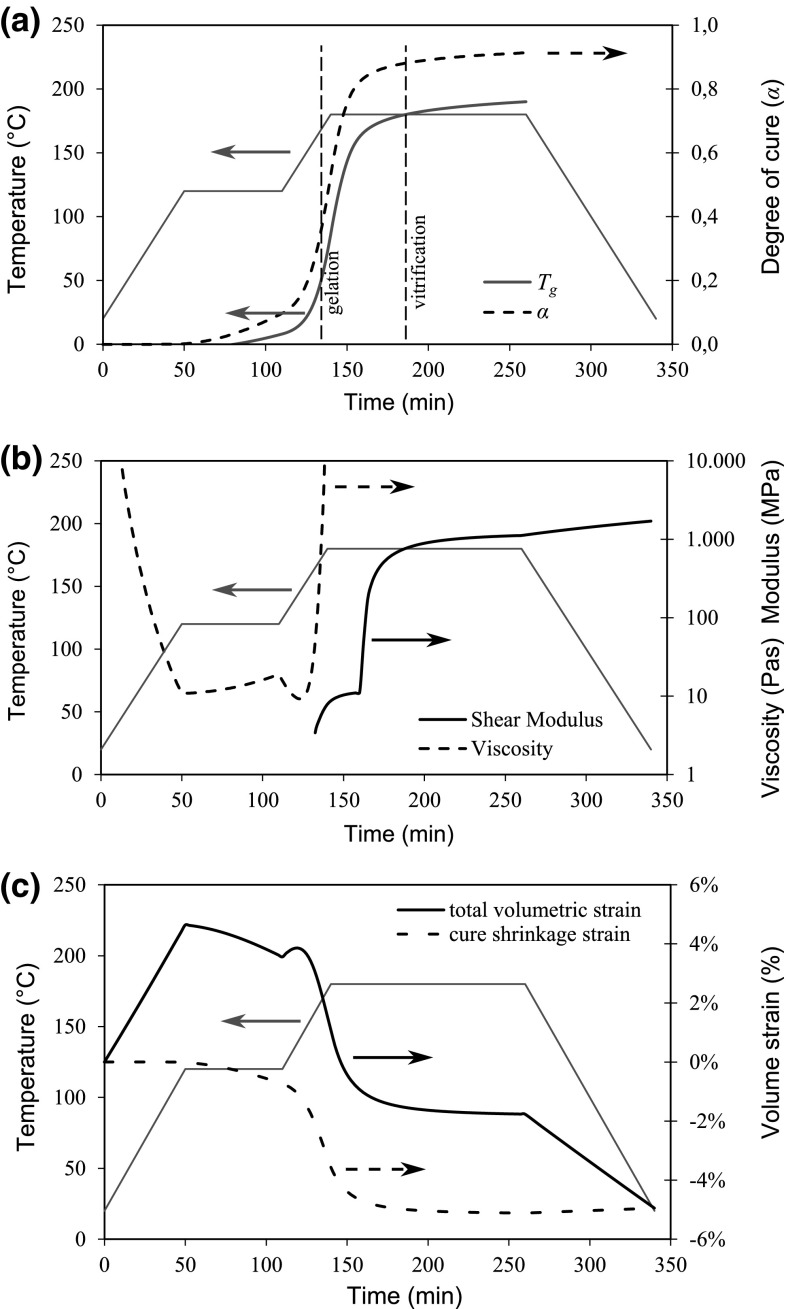
Development of properties of the resin **a** the degree of cure and the glass transition temperature, **b** viscosity and modulus, and **c** volumetric strains [13, 15, 44, 68]

Lightfoot et al. [26] tried to explain the mechanisms responsible for fiber wrinkling and fiber misalignment of unidirectional plies during lay-up of prepregs on the tool. They observed large wrinkles in parts with $90^\circ$ plies surrounded by $45^\circ$ plies when  fluoroethylenepropylene (FEP) release film was used at the tool-composite part interface. Removing the release film prevented the development of fiber wrinkles. No wrinkling was observed within the $[0]_{24}$ laminates although some in-plane waviness was detected.

In order to determine the amount of deformation resulting from the level of fiber wrinkling, a new lay-up method was recently introduced by Cinar and Ersoy [32]. In contrast to the conventional lay-up method, layers of prepreg were first laid on a flat plate, and then the whole stack was bent to conform to the surface of the L-shaped mould. This new lay-up method resulted in more fiber wrinkling in the inner surface of the parts as compared to the conventional method. The measured spring-in values were lower in the parts manufactured by the new lay-up method. The reason for this spring-in reduction was thought to be that the in-plane waviness of the fibers at the corner side helped to maintain the same arc length during curing, in turn decreasing the amount of in-plane stress and causing smaller spring-in values.

**Fig. 8:**
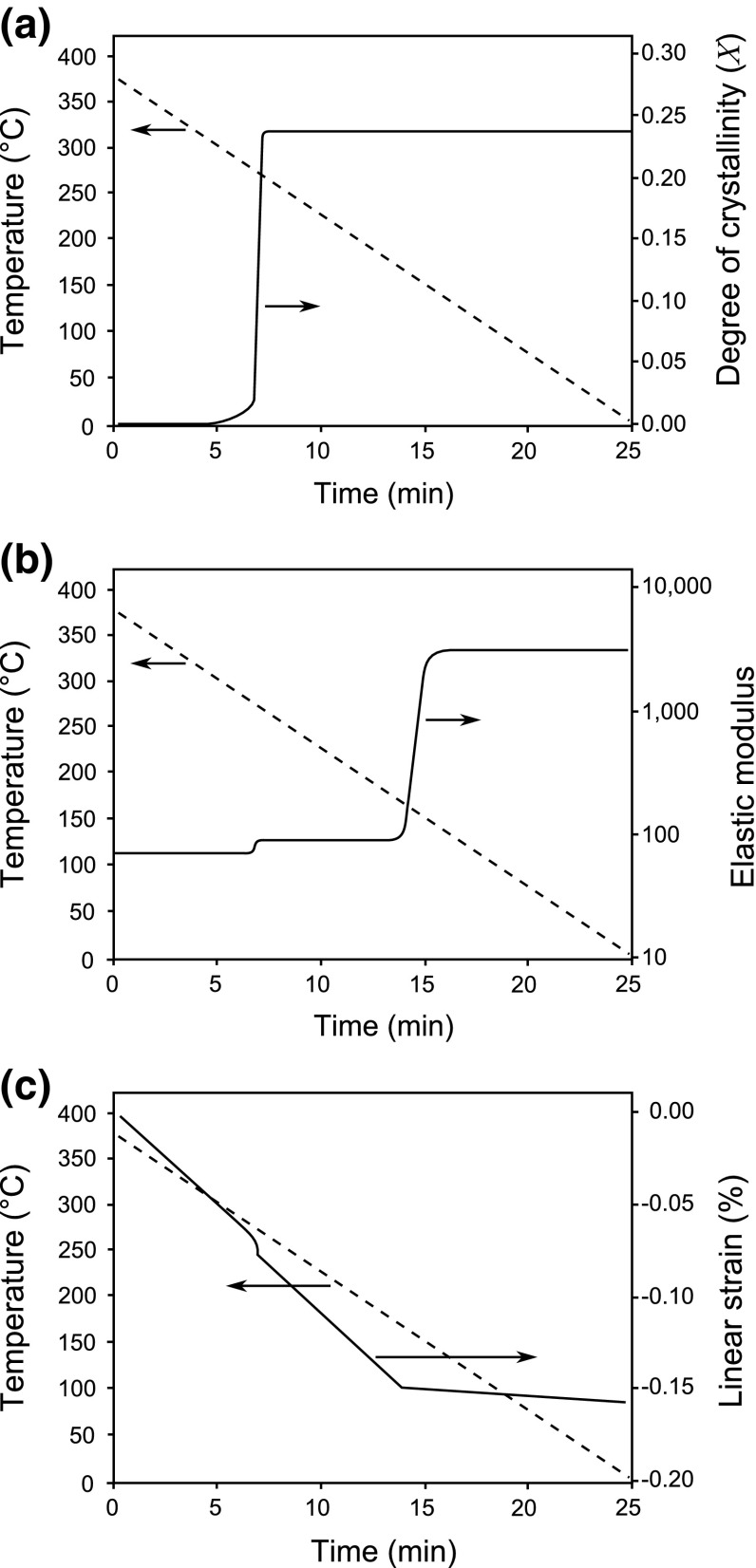
Development of properties of a PEEK thermoplastic resin: **a** the degree of crystallinity, **b** elastic modulus and **c** linear strain

### Temperature Gradients

Transient heat transfer causes thermal gradients which results in differential polymerization, shrinkage and modulus development of the matrix material in the through-thickness direction and generate residual stresses. Through-thickness temperature gradients are very small for thin parts and can be neglected but for thicker parts, rapid heat generation with the lower thermal conductivity of composite may result in significant temperature and cure gradient [34]. The evolution of macroscopic in-plane residual stresses was investigated in the thick thermoset laminates resulting from temperature and degree of cure gradients in [35–37].

**Fig. 9:**
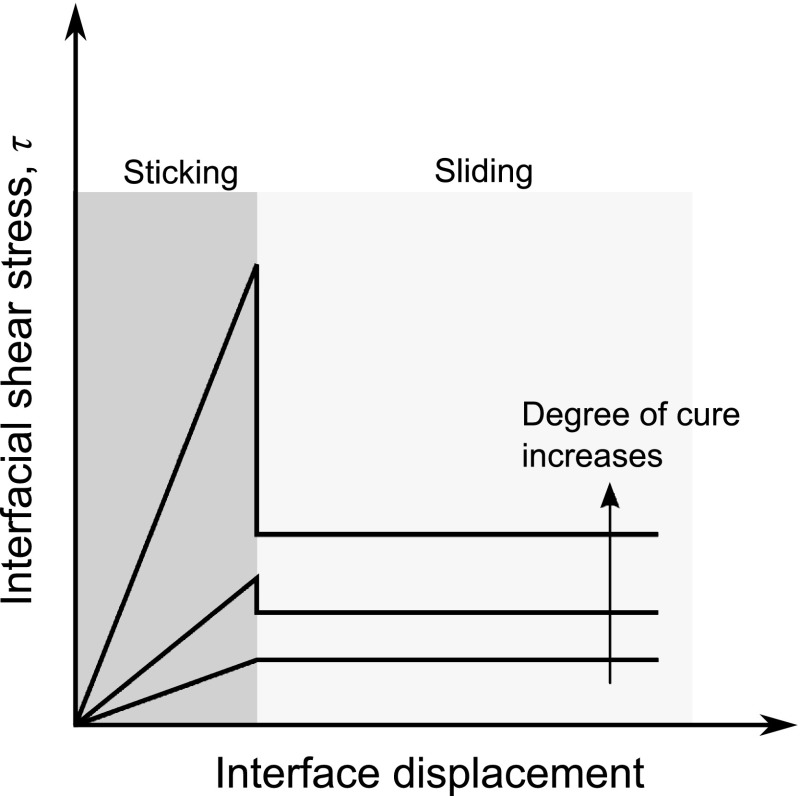
Schematic illustration of the interfacial shear stress ($\tau$) as a function of the interface displacement with sticking and sliding regions for a release agent interface [119]

Shaping and reshaping of thermoplastics based composites can take place repeatedly, since the network formation of a thermoplastic is purely physical and mainly driven by temperature [38]. In other words, a thermoplastic composite can be repetitively melted, shaped and cooled. The toughness of the thermoplastic composites are highly dependent on the cooling rates from melt temperature. Too slow cooling results in excessive crystallinity which may yield in brittle material behavior. On the other hand, a fast cooling results in low crystallinity or completely amorphous material. The temperature at which crystallisation starts also depends on the cooling rate which is difficult to control in the through-thickness direction which may cause temperature gradients in the par and should be taken into account when predicting these stresses.

## Modelling the Governing Physics

### Thermokinetics

Transient heat transfer is an important phenomenon in terms of residual stresses, since it causes thermal gradients which may result in differential vitrification or solidification as aforementioned. Transient heat transfer can in general be modelled using the same principles and approaches for both thermoplastics and thermosetting composite. One of the main differences is the exothermic heat of crystallization as the source term for heat generation in thermoplastic composites as opposed to that of curing in thermosetting resins. The status of the polymer resin changes during polymerization (curing or crystallization) in which temperature plays a crucial role. The temperature evolution during processing should therefore be evaluated using dedicated thermal models. The heat transfer equation given in Eq. 3 is solved to predict the temperature history for the fiber reinforced polymer.

3 $$\begin{aligned} \rho C_p \frac{\partial T}{\partial t} = \nabla \left( \mathbf {k} \nabla T \right) + q_{gen} \end{aligned}$$

where *T* is the temperature, $\rho$ is the density, $C_p$ is the specific heat, $\mathbf k$ is the thermal conductivity tensor and $\nabla$ is the differential operator. Generally, lumped material properties are used for composite. The source term $q_{gen}$ in Eq. 3 is related to the internal heat generation due to the exothermic reaction of polymer matrix during the process and can be expressed as [39, 40]:

4 $$\begin{aligned} q_{gen} = (1-V_f) \rho _r H_{tr}R_r(\psi , T) \end{aligned}$$

where $H_{tr}$ is the total heat of reaction, $\rho _r$ is the resin density, $V_f$ is the fiber volume fraction, $\psi$ is the degree of cure or crystallization and $R_r(\psi , T)$ is the reaction of curing or crystallization as a function of $\psi$ and *T*.

The heat transfer equation provided in Eq. 3 can be solved using two different techniques: the nodal control volume based finite element (FE) method [41] and the control volume based finite difference method [42, 43]. The temperature is solved at the nodal points at which subsequently the degree of polymerization ($\psi$) is calculated. The details of polymerization kinetics are provided in the following.

### Chemoreology

The material properties of the composite part are dependent on the degree of cure or crystallization and the temperature and they are all a function of time and location at any point in the composite part. Since the morphology of the polymer matrix is strongly affected by time and temperature as compared with the fiber material, the analysis of the mechanical properties is performed in two stages. First, the properties of the matrix material such as the modulus of elasticity, shear modulus and the Poisson’s ratio must be predicted given the degree of polymerization (curing for thermosets and crystallinity for thermoplastics) and temperature levels. The properties of the fiber reinforced composite are controlled by its fiber volume fraction and the properties of its constituents together with the fiber architecture, i.e. unidirectional (UD), mat, woven, etc. The effective properties of the fiber reinforced composite can be determined using micromechanics [35, 44–47]. Models for curing and crystallization kinetics and material properties are briefly discussed in this study.

**Fig. 10:**
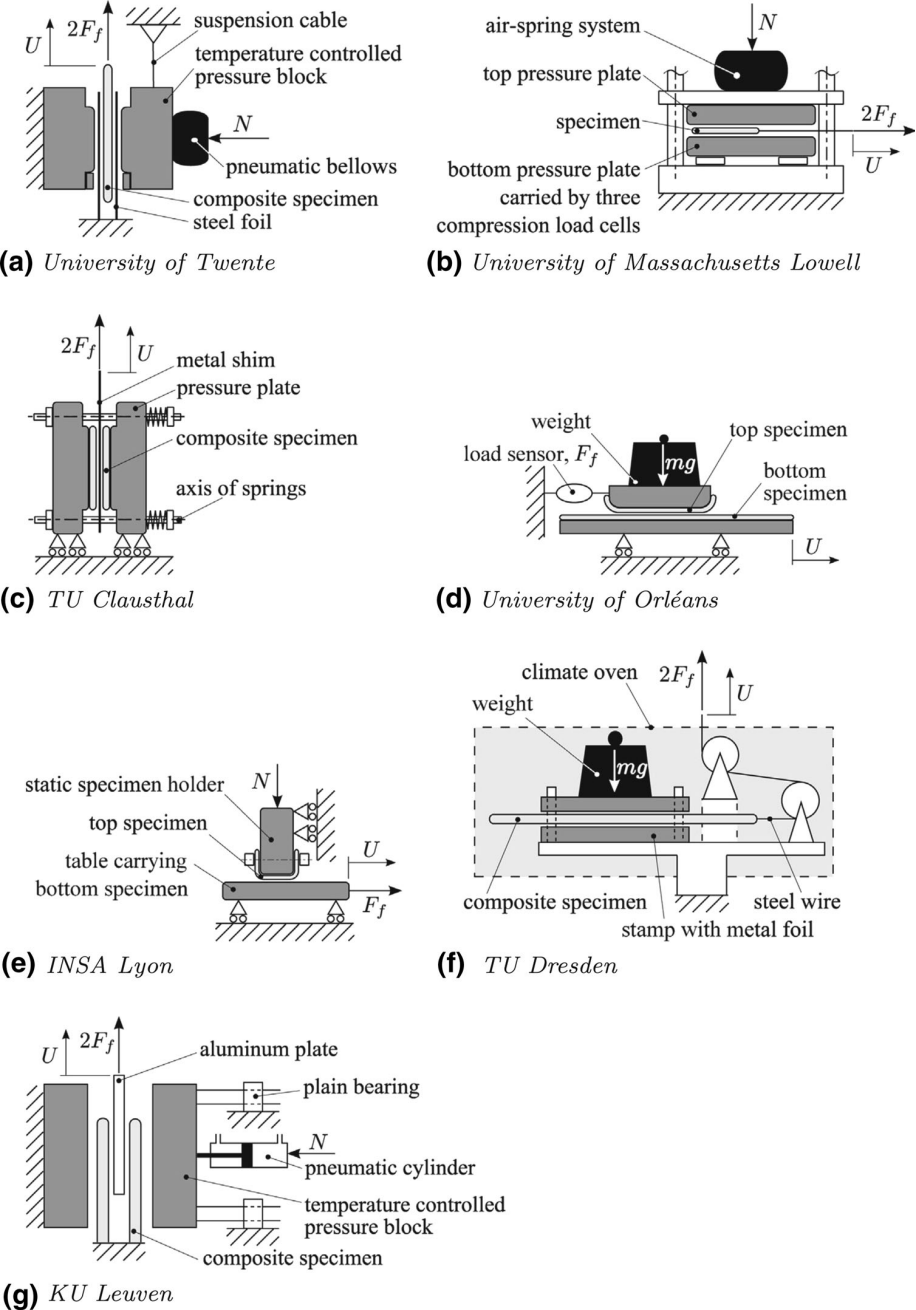
Schematic illustration of the friction test set-ups of the benchmark participants [132]

#### Thermosets

The state of the thermosetting resin is not constant during curing. There is considerable transformation from a low molecular weight monomer to a highly cross-linked polymer. The development of curing is usually defined by the degree of cure, $\alpha$ which can be determined from the ratio of the heat generated at a certain time (*H*(*t*)) during the process to the total heat generated through the complete cure ($H_{tr}$) [48] which can be expressed as:

5 $$\begin{aligned} \alpha = \frac{H(t)}{H_{tr}} \end{aligned}$$

Generally dynamic scanning calorimetry (DSC) tests are employed to determine the polymerization kinetics. Figure 5 shows the heat flow developed during the curing process of a thermosetting resin which can be measured using DSC. The area between the heat flow curve and the baseline gives the total heat generated during the exothermic curing reaction, i.e., $H_{tr}$. The baseline represents the heat required to raise the temperature. The choice of baseline is problematic, however, a straight line can be assumed. In literature, several cure kinetic models have been proposed and analyzed to describe the resin curing polymerization [49–57]. In general, Arrhenius-type equations are employed for most of the cure kinetics models. An example of a well-known semi-empirical autocatalytic model [34, 58, 59] is expressed as:

6 $$\begin{aligned} R_r(\alpha ,T) = \frac{d\alpha }{dt} = A_{0}\exp \left( \frac{-E_a}{RT}\right) \alpha ^m(1-\alpha )^n \end{aligned}$$

where $A_0$ is the pre-exponential constant, $E_a$ is the activation energy, *R* is the universal gas constant and *m* and *n* are the orders of reaction (kinetic exponents). On the other hand, $n^{\mathrm{th}}$-order cure models are particularly used for epoxy systems [60, 61] since they experience no autocatalyzation. The corresponding expression is given as:

7 $$\begin{aligned} R_r(\alpha ,T) = \frac{d\alpha }{dt} = A_{0}\exp \left( \frac{-E_a}{RT}\right) (1-\alpha )^n \end{aligned}$$

The glass transition temperature $T_g$ is an another important property, where the matrix material transforms from a soft rubbery state to a hard glassy state. The evolution of the $T_g$ is generally modelled as a function of the degree of cure ($\alpha$) using the Di Benedetto equation [62, 63] and expressed as:

8 $$\begin{aligned} \frac{T_g-T_{g0}}{T_{g\infty }-T_{g0}} = \frac{\lambda \alpha }{1-(1-\lambda )\alpha } \end{aligned}$$

where $T_{g0}$ and $T_{g\infty }$ are the glass transition temperatures of uncured and fully cured resin, respectively and $\lambda$ is a constant used as fitting parameter [63]. Moreover, the dependence of glass transition on the degree of cure can also be estimated using experimental data and the corresponding relation is given as [34, 64]:

9 $$\begin{aligned} T_g = T_g^0 + a_{Tg}\alpha \end{aligned}$$

where $T_g^0$ is the glass transition temperature at $\alpha = 0$ and $a_{Tg}$ is a constant.

The rheological behaviour of the processing resin system directly affects the viscosity during the process. The viscosity ($\eta$) can be modelled as a function of temperature and degree of cure as written: [57]:

10 $$\begin{aligned} \eta (\alpha ,T) = \eta _\infty \exp \left( \frac{\Delta E_{\eta }}{RT} + K\alpha \right) \end{aligned}$$

where $\Delta E_{\eta }$ is the viscous activation energy, $\eta _{\infty }$ is the initial viscosity, *K* is a constant, *R* is the universal gas constant, *T* is the absolute temperature. A rheometer is utilized to measure the viscosity as a function of time and temperature. Subsequently, a least squares non-linear regression analysis can be performed upon the measured data in order to determine the constants in the viscosity model [57]. The gelation is defined as the point at which the state of the resin changes from a viscous liquid to a rubbery gel. As reported in [65], the gelation occurs when the viscosity of the resin increases to infinity.

#### Thermoplastics

When a thermoplastic polymer is cooled from a molten state, the random liquid structure may partially transform to an ordered periodic crystalline one. This process is called crystallization. The transformation is possible only above the glass transition temperature, $T_g$, but below the melting temperature, $T_m$. Thermoplastic polymers, having long molecular chains and very high viscosity, cannot completely crystallize. Many of them solidify into amorphous solids while others form semi-crystalline structures. In terms of process modeling, the cooling step is the step where the modulus of the resin, hence residual stresses develop during cooling from molten state, as opposed to thermosetting resins where curing starts at early stages, during heat-up ramps and completes when $T_g$ of the resin reaches the process temperature.

**Fig. 11:**
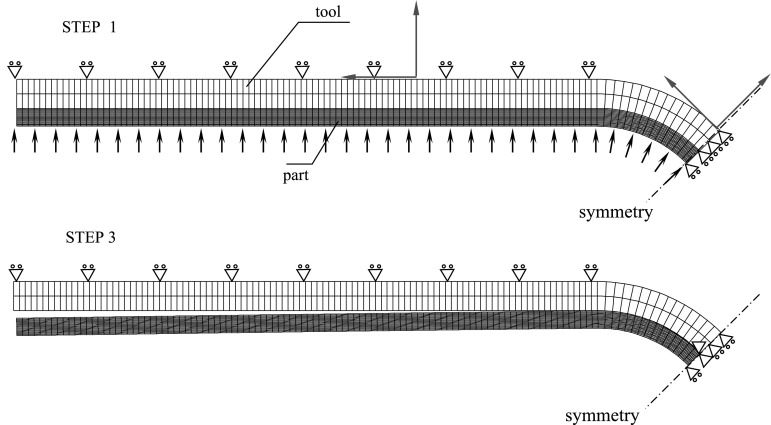
The FE mesh and boundary conditions at the 1st, 2nd and 3rd step [108]

A complete characterization of the morphology of thermoplastics should include many details of internal structure such as shape, size and number of spherulites and crystallites. However, the overall crystallinity is usually used as a macroscopic representation of internal structure and is defined as the ratio of the mass of the crystalline phase ($m_c$) to the total mass ($m_t$):

11 $$\begin{aligned} X_{mc} = \frac{m_c}{m_t} \end{aligned}$$

or, as the ratio of the crystalline volume ($V_c$) to the total volume ($V_t$):

12 $$\begin{aligned} X_{vc} = \frac{V_c}{V_t} \end{aligned}$$

The former is called the mass fraction crystallinity ($X_{mc}$) and the latter the volume fraction crystallinity ($X_{vc}$). They are related to each other by:

13 $$\begin{aligned} X_{vc}= \, & {} \frac{X_{mc}/\rho _c}{X_{mc}/\rho _c + (1-X_{mc})/\rho _a} \end{aligned}$$

14 $$\begin{aligned} X_{mc}= \, &{} \frac{X_{vc}\rho _c}{X_{vc}\rho _c + (1-X_{vc})\rho _a} \end{aligned}$$

where $\rho _c$ is the density of the crystalline phase and $\rho _a$ is the density of the amorphous phase.

DSC is the most commonly used technique of determining crystallinity, but it has low reproducibility and accuracy, especially in estimating crystallinity levels of fast-cooled specimens [69].

As explained in Sect. 3.2.1 for thermosets, a DSC device measures the heat flow necessary to achieve a temperature change. It can also be used for both isothermal and nonisothermal crystallization experiments for thermoplastics. A sample is heated from room temperature to above the melting temperature in the DSC. For isothermal crystallization experiments, it is suddenly cooled from melt temperature to the desired temperature and the heat flow is monitored. For a non-isothermal crystallization experiment the sample is cooled from melt temperature at the desired cooling rate and the heat flow is monitored. The heat flow curve as a function of time in Fig. 5 represents a typical DSC trace for a thermoplastic matrix material. Since the crystalline phase represents a lower state of energy, the formation of crystalline material reflects itself as an exothermic peak on the heat flow curve. The area between the peak and the baseline gives the latent heat of melting.

**Fig. 12:**
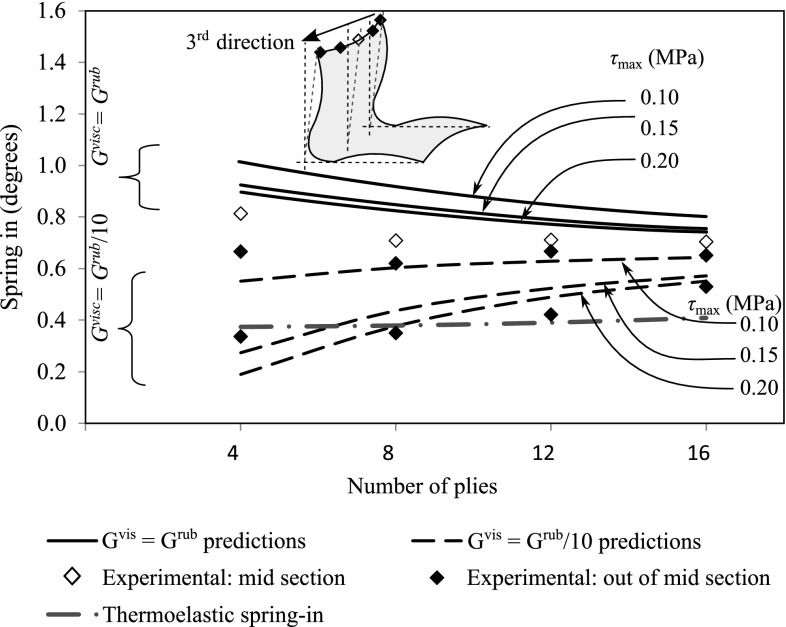
Effect of sliding shear stress and viscous shear modulus on spring-in predictions for UD-R25 parts [108]

A composite manufacturing process can to some extent be physically simulated using a DSC device, and the crystallinity and crystallization rate at any instant of the process can be calculated. However, it should be noted that the maximum rate of cooling or heating that could be achieved by the DSC equipment is usually below the requirements of the process. The degree of crystallinity as a function of time can be found from DSC scans by using the following equation:

15 $$\begin{aligned} X_{mc} = \frac{H(t)}{(1-X_{mr})H_f} = \frac{\int _0^t \dot{Q}(t)dt}{(1-X_{mr})H_f} \end{aligned}$$

where $X_{mr}$ is mass fraction reinforcement, $\dot{Q}(t)$ is the flow rate, *H*(*t*) is the heat flow until the time *t* as defined in Fig. 5 and $H_f$ is the enthalpy of fusion of fully crystalline material. $H_f$ can be obtained from an extrapolation between the melting enthalpy of various thermoplastic samples and crystallinity determined by Wide Angle X-Ray Diffraction. Eq. 15 is applicable to the neat polymer by assuming $X_{mr}=0$. The mass and volume crystallinity levels are related by Eqs. 13 and 14. The maximum in the heat flow curves represents the changeover from the faster, primary process of crystallization to a slower, secondary process due to the impingement of growing spherulites. The construction of a crystallinity versus time plot using the DSC trace of the heat flow versus time is the basis for the models proposed for crystallization kinetics.

Most of the available phase transformation models originate from the work done by Avrami [70–72]. It is based on the assumption that the new phase is nucleated by germ nuclei, which already exist in the old phase. As the transformation proceeds, the germ nuclei diminish as some of them become growth nuclei for the new phase and ingest others while growing. Furthermore, the linear growth rate of a crystal from a growth nucleus is assumed to be constant.

**Fig. 13:**
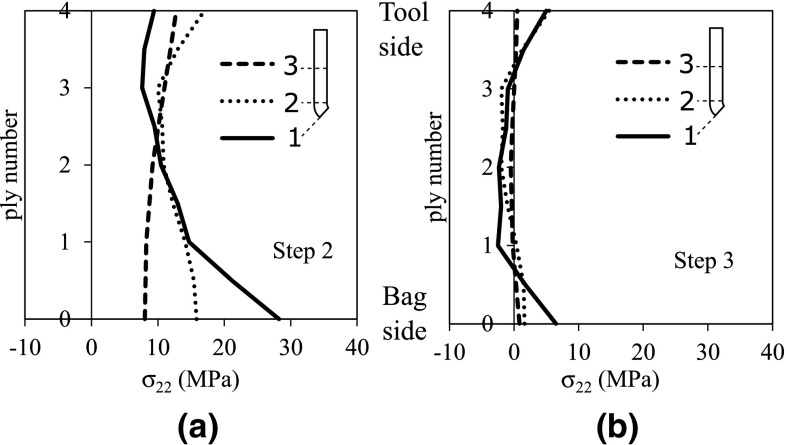
Predicted residual stresses in the fiber direction for UD4-R15 part at the end of **a** $2^{\mathrm{nd}}$ step and **b** $3^{\mathrm{rd}}$ step. [109]

It was proposed in [73, 74] that the crystallization kinetics of PEEK resin and its composites could be analyzed according to an Avrami type analysis. The basic isothermal Avrami expression provides:

16 $$\begin{aligned} X_{vc} = X_{vc}^{\infty }(1-\exp (-K(T)\cdot t^n)) \end{aligned}$$

where *K*(*T*) is the crystallization rate constant, *n* is the Avrami exponent, *t* is the time, *T* is the temperature [K], $X_{vc}$ is the volume fraction crystallinity, and $X_{vc}^\infty$ is the equilibrium volume fraction crystallinity.

A model based on a single mechanism was used in Cebe et al. [73]; however, in order to fit the Avrami equation to experimental data, they have used different *n* values (between 2.8 to 3.3) for different crystallization temperatures. It was found in [74] that the crystallization process of PEEK followed a dual mechanism, one corresponding to an Avrami exponent of 2.5 and the other corresponding to an Avrami exponent of 1.5. Since PEEK is one of the most commonly used thermoplastics in composites due to its benefits related to strength, toughness and elevated temperature properties, the development of its properties during processing is extensively investigated in the literature. In this review, we are going to adopt this material as a model material and review the literature related to PEEK.

**Fig. 14:**
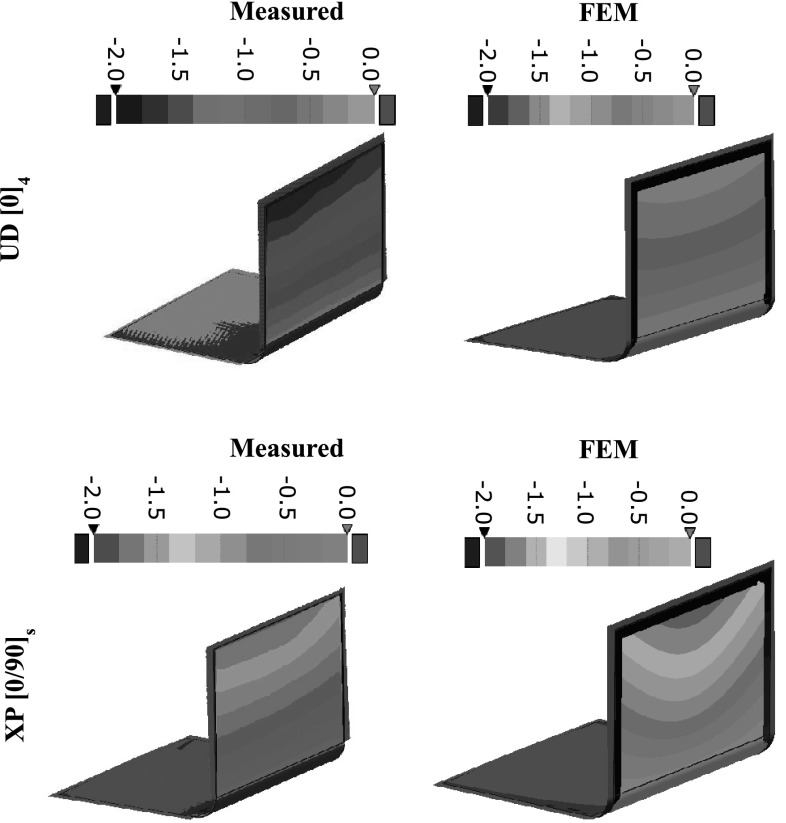
Measured and predicted deformation patterns for $[0]_4$ and $[0/90]_s$ L-section parts with R15 mm corner radius [109]

Thus, a model was proposed in [74] to describe the crystallization kinetics of crystallization of PEEK that involves two competing nucleation and growth processes, based on a linear combination of the two Avrami expressions. They used an integral type Avrami expression to describe the non-isothermal crystallization conditions.

The complete expression for their kinetic model is [74]:

17 $$\begin{aligned} \frac{X_{vc}}{X_{vc}^\infty } = w_1 F_{vc1} + w_2 F_{vc2} \end{aligned}$$

where

18 $$\begin{aligned} w_1 = 1-w_2 \end{aligned}$$

$F_{vci}$ is defined for isothermal crystallization as:

19 $$\begin{aligned} F_{vci} = 1-\exp (-K_i(T)\cdot t^{n-1}) \qquad \text {for} \qquad i=1,2 \end{aligned}$$

and for non-isothermal crystallization as:

20 $$\begin{aligned} F_{vci} = 1-\exp \left[ -\int _0^t K_i(T) n_i t^{n_i-1} dt \right] \qquad \text {for} \qquad i=1,2 \end{aligned}$$

where

21 $$\begin{aligned} K_i(T) = -C_{1i}T\exp \left[ - \left( \frac{C_{2i}}{T-T_g+51.6} + \frac{C_{3i}}{T(T_{mi}-T)^2} \right) \right] \quad \text {for} \quad i=1,2 \end{aligned}$$

where $K_i(T)$ is the crystallization rate constant, $F_{vci}$ are the normalized volumes fraction of crystallinities, $T_{mi}$ are the crystal melt temperatures, $n_i$ are the Avrami exponents, $C_{1i}$, $C_{2i}$ and $C_{3i}$ are the model constants and $w_i$ are the weight factors for dual mechanisms ($i=1,2$). These relationships can be used to describe the crystallization and growth processes. Values for the weight factors $w_1$ and $w_2$ provide the relative importance between two competing mechanisms.

### Chemical Shrinkage

Crystallization shrinkage can be handled in the same way as in cure shrinkage to calculate the shrinkage strains in main material directions in continuous fiber composites by using micromechanics. Another main difference is related to the material models, which depend, besides temperature, on degree of crystallinity in thermoplastic composites and degree of cure in thermosetting composites. The thermal expansion/contraction and chemical shrinkage behavior of the composite are determined using a micromechanics model.

**Fig. 15:**
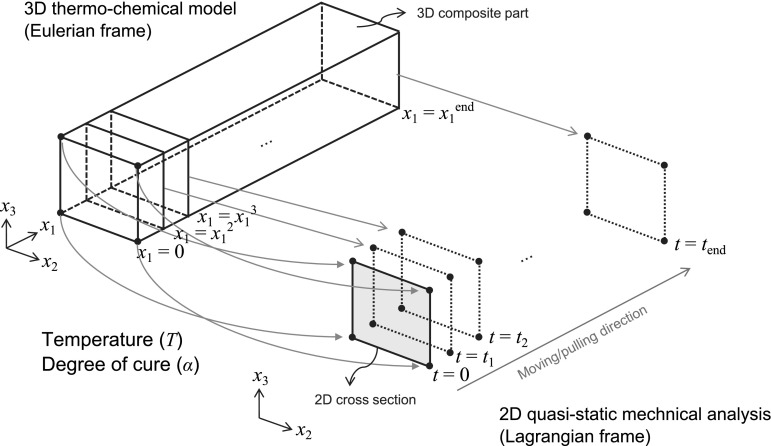
Representation of the sequentially coupled 3D thermo-chemical model with a 2D quasi-static mechanical model for pultrusion processes [152]

The chemical shrinkage of a thermosetting resin can be expressed via the total volumetric shrinkage ($V_{sh}$) as explained in the following. Assuming a uniform contraction for a unit cell in the resin, the isotropic incremental resin shrinkage strain ($\dot{\varepsilon }_{r}^c$) is calculated for a thermosetting matrix material as [35]:

22 $$\begin{aligned} \dot{\varepsilon }_r^{c} = \root 3 \of {1+\Delta \alpha \cdot V_{sh}} - 1 \end{aligned}$$

where $\Delta \alpha$ is the change in degree of cure $\alpha$ and $V_{sh}$ is the total volumetric shrinkage of the thermosetting resin system. The shrinkage of the resin starts after the gelation point and follows a linear relationship with the degree of cure [63].

The volume change of the polymeric resin during curing can be measured by various methods. Cure shrinkage strains can be calculated from measurements of the change in the dimensions of a test specimen during a cure. White and Hahn [75] used a simple method to measure the cure shrinkage. Prepreg plies were placed in a computer controlled oven and cured according to the MRCC. During the cure cycle, at each of the predetermined points, a prepreg sample was taken out of the oven and the thickness was measured after cooling to room temperature. The cure strains were then calculated from the changes in dimensions of the samples at room temperature. Another common method is to directly measure the chemical shrinkage using a volumetric dilatometer [76–79] or a thermo-mechanical analyser (TMA) [34]. Russell et al. [76] used a PVT apparatus to measure the chemical shrinkage of neat 3501-6 resin. The volume change in the resin was determined by the deflection of a bellows filled with mercury that was covering a piezometer cell. A Linear Variable Differential Transducer (LVDT) measured the deflection of the bellows at the thickness direction. Johnston [34] used a Thermo-Mechanical Analyser to measure the shrinkage strains by monitoring the displacement of a small probe pressing lightly on the specimen surface. All specimens were processed isothermally (130, 150, and $170\,{}^\circ \hbox {C}$) to eliminate thermal strain effects. A non-contact technique was used by Garstka [15] to measure cure shrinkage strains of unidirectional and cross-ply laminates. A non-interlaced camera was mounted on a tripod and focused on contrasting targets marked on the sides of the two steel plates that were in contact with the composite specimen during the test. It was found that cross-ply laminates have more through-thickness chemical shrinkage than unidirectional ones due to the constraints imposed by fibers in both in-plane directions in the cross-ply specimens. Ersoy and Tugutlu used a dynamic mechanical analyser (DMA) in compression mode to find the through-thickness cure shrinkage strain of partially cured unidirectional and cross-ply composite samples [48]. The experimental measurements showed the through-thickness cure shrinkage strains in cross-ply samples to be twice of the strains in unidirectional ones, which is similar to the findings in [15]. The concept of an equivalent CTE was used to account for resin shrinkage during the curing process [25, 35, 80, 81]. The volumetric cure shrinkage of the resin was implemented by changing the CTE of the composite material in numerical models.

**Fig. 16:**
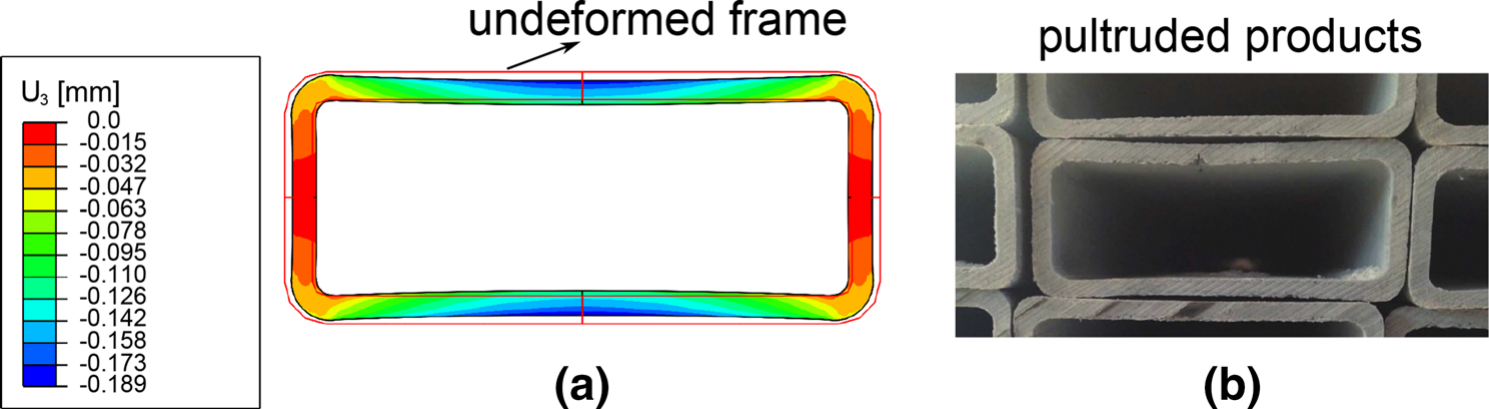
The predicted residual warpage (**a**) and the pultruded products with warpage (**b**). Note that the full section is depicted for the calculated deformed geometry with a scaling factor of 5 (**a**) [158]

The existing analytical approaches that used Eq. 2 to predict spring-in of curved parts do not distinguish between cure shrinkage in different phases of the cure, though material properties like the shear modulus of the resin change during curing. Wisnom et al. [82] examined the effect of material properties in the rubbery state on the final spring-in value by adding another term to Eq. 2 which takes into account the rubbery shear modulus of the composite. It was found that the spring-in values were smaller as compared to the values found directly from Eq. 2. Due to the low shear modulus of resin, there is some shear deformation (shear-lag) between the plies to maintain the same arc length during curing. This decreases the amount of in-plane stress and causes smaller spring-in values, as shown schematically in Fig. 6. The shear-lag phenomenon also shows its effect in a two-step FE model that predicts the spring-in angles in C-sections [83]. The predicted and measured spring-in values for different thicknesses show a considerable decrease in spring-in with part thickness, which matches well the trend predicted from the analytical study of Wisnom et al. [82]. It can be concluded from these studies that the effect of cure shrinkage on spring-in decreases with increasing part thickness due to this shear-lag phenomenon.

In thermoplastic materials, the shrinkage strain of the resin can directly be calculated from the degree of crystallinity if the densities of the amorphous and crystalline regions are known. The isotropic crystallization shrinkage strain, $\dot{\varepsilon }_r^{c}$, can be related to the incremental volumetric resin shrinkage strain due to crystallization of a unit volume element of resin, $\Delta V_c$, by [84]:

23 $$\begin{aligned} \dot{\varepsilon }_r^{c} = \frac{-1+\sqrt{1+(4/3)\Delta V_c}}{2} \end{aligned}$$

The incremental volumetric resin shrinkage strain due to crystallization, $\Delta V_c$, can be computed from the ratios of the instantaneous, crystallinity dependent resin densities at each time increment as [84]:

24 $$\begin{aligned} \Delta V_{c} = \frac{\rho (X_{vc})^{n+1} - \rho (X_{vc})^n}{\rho (X_{vc})^n} \end{aligned}$$

where $\rho (X_{vc})^{n+1}$ is the resin density at the next time step $n+1$, and $\rho (X_{vc})^{n}$ is the resin density at the present time step *n*. The resin density at any time increment is determined using a “rules of mixtures” formulation from the densities of the amorphous ($\rho _{am}$)and crystalline phases ($\rho _{cr}$) and the instantaneous crystallinity as:

25 $$\begin{aligned} \rho (X_{vc}) = X_{vc}\rho _{cr} + (1-X_{vc})\rho _{am} \end{aligned}$$

### Modelling of Compaction

The manufacturing process of fiber reinforced composite materials can be modelled by taking into account the three states of the resin. These are the viscous, rubbery and glassy states. In the viscous state, the viscosity of the resin is low and it flows in response to pressure gradients in the laminate. The impregnation of the fibers has been modelled using a two dimensional (2D) flow model for composite materials [85, 86] based on Darcy’s law for flow in porous medium. The Darcian flow theory was coupled with a stress formulation in these studies. Generally, composite structures have one dimension that is much larger than other two dimensions, justifying the use of a plane strain condition in the model [85, 86]. The resin was assumed to be an incompressible Newtonian fluid (for simplicity).

**Fig. 17:**
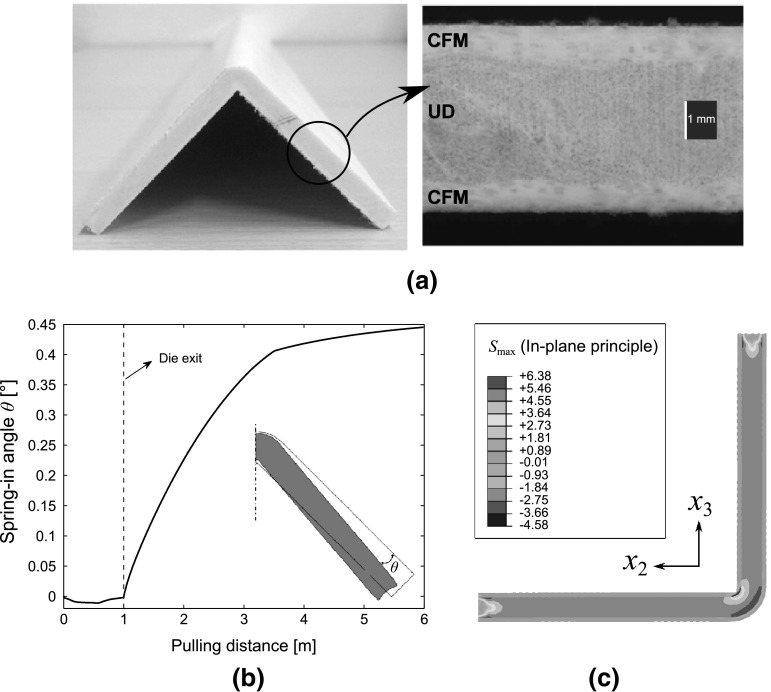
**a** Pultruded L-shaped profile and corresponding cross section showing the CFM and UD roving layers. **b** The development of the spring-in angle ($\theta$) inside the heating die and post die regions. **c** The predicted maximum principle stress ($S_{\max }$) distribution at the end of the process. Note that $x_2$ and $x_3$ are the directions transverse to the pulling direction. [159]

A number of models have been developed to predict composite resin flow in autoclave processing [34, 85–91]. These studies were primarily focused on the consolidation of simple shaped laminates. Gutowski et al. [88] proposed a squeezed-sponge 3D flow and a 1D compaction model, and considered the composites as a deformable unidirectional fiber reinforcement system where the load is balanced by the average resin pressure and the average effective stress in the fiber network. Darcy’s Law in a porous medium was used for flow in the vertical direction of the composite material. Dave et al. [87] used the same approach but their model considered the flows in different directions to be coupled. Hubert et al. [85] and Li and Tucker [90] developed a 2D flow-compaction model for L shaped composite laminates. They solved the equations using a finite element method. Hubert et al. [85] used an incremental, quasi linear elastic model for the solid bed stress. On the other hand, Li and Tucker [90] developed a special hyper-elastic model for fiber bed stress where the mesh geometry and fiber orientation was updated as consolidation proceeded. Li and Tucker [90] also observed the fiber buckling effect in their numerical analysis.

## Constitutive Material Modelling

In this section different constitutive modelling approaches which have been commonly used in literature are presented namely i) the linear elastic model, ii) the viscoelastic model and iii) the path dependent model. The stress-strain relations are presented together with the elastic modulus of the matrix material. It should be noted that the effective mechanical properties as well as the thermal and chemical shrinkage strains for the composite part are calculated using the micromechanics approaches as aforementioned.

### Linear Elastic Model

#### Thermosets

The stiffness of the resin significantly depends on the degree of cure ($\alpha$). The cure dependent instantaneous isotropic resin modulus ($E_r$) was proposed in [35] in which the curing process was divided into three distinct regions. In the first and third region, the resin modulus is constant, while the resin modulus is considered to be a function of degree of cure in the second region. In the first region, the resin was fully uncured and assumed to behave as a viscous fluid. In the second region, the stiffness of the resin significantly increased and the specific volume of the resin decreased due to chemical shrinkage. In the last region, no further chemical shrinkage occurs. The elastic modulus of the matrix material was defined as a function of cure degree and expressed as [35]:

26 $$\begin{aligned} E_r = (1-\alpha _{mod})E_r^{0} + \alpha _{mod} E_r^\infty + \gamma \alpha _{mod}(1-\alpha _{mod})(E_r^{\infty }-E_r^{0}) \end{aligned}$$

and

27 $$\begin{aligned} \alpha _{mod} = \frac{\alpha -\alpha _{mod}^{gel}}{\alpha _{mod}^{diff}-\alpha _{mod}^{gel}} \end{aligned}$$

where $E_r^0$ and $E_r^{\infty }$ are the fully uncured (first region) and fully cured (third region) resin moduli, respectively, $\alpha _{mod}^{gel}$ and $\alpha _{mod}^{diff}$ are the bounds on the degree of cure between which resin modulus is assumed to develop (second region) and $\gamma$ is a parameter representing the competing mechanisms between stress relaxation and chemical hardening [35]. It should be noted that $E_r^0$ is generally assumed to be $E_r^{\infty }/1000$ as a first approximation [35, 64, 92]. Eq. 26 has been modified by incorporating the temperature dependency as suggested in the CHILE approach [34, 58] which exhibits the cure hardening and also thermal softening as shown in Eq. 28.

28 $$\begin{aligned} E_r = \left\{ \begin{array}{lll} E_r^{0} &{} &{} T^* \le T_{C1} \\ \displaystyle E_r^{0} + \frac{T^{*}-T_{C1}}{T_{C2}-T_{C1}}(E_r^{\infty }-E_r^{0}) &{} for &{} T_{C1} < T^{*} < T_{C2} \\ E_r^{\infty } &{} &{} T_{C2} \le T^{*} \end{array}\right. \end{aligned}$$

where $T_{C1}$ and $T_{C2}$ are the critical temperatures at the onset and completion of the glass transition, respectively and $T^*$ represents the difference between the instantaneous glass transition temperature $T_g$ and the temperature *T* of the resin, i.e. $T^* = T_g - T$ [34, 58].

**Fig. 18:**
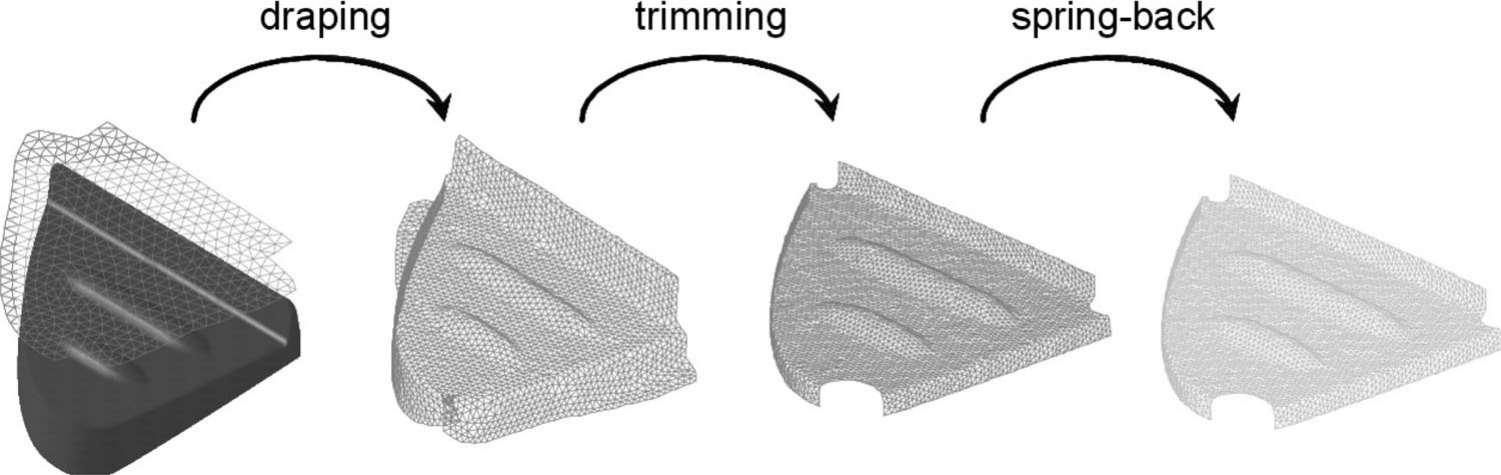
Modelling approach for the rubber press forming of an Airbus A-380 wing leading edge stiffener made of 8H-satin glass and PPS. From *left* to *right*: lower steel tool + laminate preform, drape result, trimming result and the deformed geometry after spring-back prediction [183]

Figure 7 shows the development of material properties for a typical thermosetting resin (Hexcel’s 8552 epoxy resin) [68], i.e., the development of the degree of cure ($\alpha$), the glass transition temperature and the viscosity together with the shear modulus and the volumetric strain. A sharp rise in the degree of cure and viscosity during the second ramp of the cure cycle can be seen around the gel point, $\alpha = 0.30$ indicating a state change from viscous fluid to a rubbery solid at which the resin exhibits an elastic modulus and is capable of sustaining mechanical load. Vitrification occurs approximately 45 min after the second hold period where the degree of cure value is around 0.7 at which $T_g$ reaches the process temperature and the elastic modulus starts rising significantly since the resin state changes from a rubbery to a glassy solid. As the resin passes through the gelled state, a considerable volumetric shrinkage occurs which is an important source of residual stresses [68].

#### Thermoplastics

Ply properties in both the longitudinal and transverse directions as a function of time, temperature and crystallinity are required to evaluate the state of residual stress. These properties are highly dependent upon the individual constituent properties and the volume fraction of the constituent materials.

In the case of semi-crystalline matrix composites like APC-2, the properties of the matrix are determined by the properties of its amorphous and crystalline phases and the instantaneous volume fraction of crystallinity.

The standard linear solid (SLS) kinetic-viscoelastic model was proposed in [84] to predict the matrix material properties. According to this model the dynamic mechanical storage flexural compliance $S^\prime$ and loss flexural compliance $S^{\prime \prime }$ consisting of amorphous ($S_{am}^\prime$, $S_{am}^\prime \prime$) and crystalline ($S_{cr}^\prime$, $S_{cr}^\prime \prime$) contribution are expressed as a function of crystallinity:

29 $$\begin{aligned} S^\prime= \, & {} S_{am}^\prime (1-X_{vc}) + S_{cr}^\prime (X_{vc}) \end{aligned}$$

30 $$\begin{aligned} S^{\prime \prime }= \, & {} S_{am}^{\prime \prime } (1-X_{vc}) + S_{cr}^{\prime \prime } (X_{vc}) \end{aligned}$$

The amorphous contribution undergoes a viscoelastic relaxation according to the following relations:

31 $$\begin{aligned} S^\prime _{am}= \, & {} S_{ua} + (S_{ra}-S_{ua})[cos(\varphi )]^a cos(\varphi a) \end{aligned}$$

32 $$\begin{aligned} S^{\prime \prime }_{am}= \, & {} (S_{ra}-S_{ua})[cos(\varphi )]^a sin(\varphi a) \end{aligned}$$

33 $$\begin{aligned} \varphi= \, &{} \arctan (w \tau _{am}) \end{aligned}$$

where $S_{ua}$ is the unrelaxed amorphous compliance, $S_{ra}$ is the relaxed amorphous compliance, *w* is the angular frequency, *a* ranges from 0 to 1, and accounts for shifting the maximum of the dynamic mechanical loss modulus due to degree of crystallinity, and $\tau _{am}$ is the amorphous retardation time. The crystalline storage compliance was assumed to be constant and the crystalline loss compliance was assumed to be zero due to the fact that the crystalline contribution does not undergo relaxation:

34 $$\begin{aligned} \begin{array}{l} S^{\prime }_{cr} = S_{uc} \\ S^{\prime \prime }_{cr} = 0 \end{array} \end{aligned}$$

where $S_{uc}$ is the unrelaxed crystalline compliance.

The temperature dependency of the retardation time was determined by two approaches: (i) the Andrade (Arrhenius) approach and (ii) The Williams, Landel, and Ferry (WLF) approach. Below the glass transition temperature, the Arrhenius approach was used. The resulting expression for time-temperature superposition of the retardation time is given by:

35 $$\begin{aligned} \tau = \tau _0 \exp \left[ \frac{E_a}{R} \left( \frac{1}{T} - \frac{1}{T_0} \right) \right] \end{aligned}$$

with the reference temperature, $T_0$, corresponding to the reference retardation time $\tau _0$. The activation energy and the universal gas constant were represented by $E_a$ and *R*, respectively. The WLF approach to be used above the glass transition temperature with time-temperature shifting for the retardation time was given according to the expression:

36 $$\begin{aligned} \log \left( \frac{\tau _{am}}{\tau _0} \right) = \frac{-C_1 (T-T_0)}{C2 + (T-T_0)} \end{aligned}$$

where $C_1$ and $C_2$ are constants determined from experimental time-temperature superposition.

**Fig. 19:**
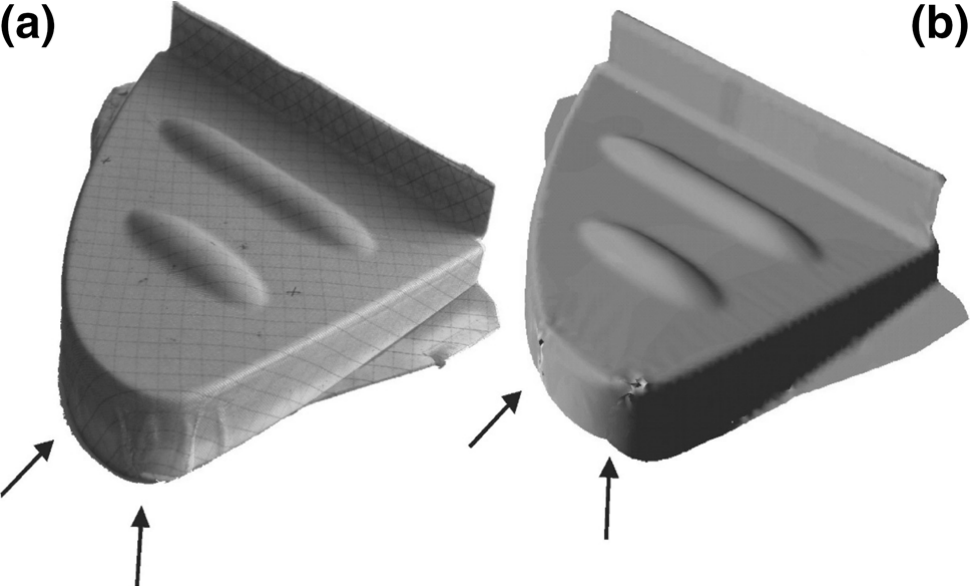
Wrinkle formation after draping: **a** experimental and **b** predicted shape. The *arrows* indicate the locations of wrinkles and folds [183]

It was noted in [84] that the temperature dependency of the retardation time was much more difficult to determine above the glass transition temperature for semicrystalline polymers due to recrystallization of the amorphous material. They performed stress relaxation and creep experiments up to the recrystallization temperature of PEEK (approximately $170^\circ \hbox {C}$). They extended the WLF time-temperature superposition to $+50^\circ \hbox {C}$ by extrapolation of the amorphous data. Above $+50^\circ \hbox {C}$ and below $-50^\circ \hbox {C}$ the asymptotic values of the unrelaxed and relaxed storage compliance were utilized. They assumed that these values were independent of temperature.

Dynamic mechanical measurements were performed on neat PEEK films showing an increase in the temperature of the maximum in the loss modulus with increasing degree of crystallinity. Their viscoelastic model accounts for this change by varying *a* in Eqs. 31 and 32 and the reference temperature. Dynamic mechanical data were fitted to noncrystalline and equilibrium crystalline modulus values and assumed to vary linearly between these two extremes. The SLS model was able to fit experimental dynamic data for a wide range of cooling histories.

The dynamic mechanical storage and loss compliance were related to the matrix modulus, $E_r$, through the storage modulus $E^\prime$ by:

37 $$\begin{aligned} E_r \approx E^\prime = \frac{S^\prime }{S^{\prime 2} + S^{\prime \prime 2}} \end{aligned}$$

Assuming the Poisson’s ratio of the matrix ($\nu _r$) being constant, the matrix modulus computed over each time increment is related to the instantaneous resin shear modulus ($G_r$) based on the following isotropic material relation:

38 $$\begin{aligned} G_r = \frac{E_r}{2(1+\nu _r)} \end{aligned}$$

During each time increment in the process simulation, the instantaneous resin properties were used to compute effective mechanical properties in the composite through the micromechanics model as aforementioned.

The SLS model calculates the elastic modulus of the resin as a function of temperature and degree of crystallinity and in this sense it is very similar to the CHILE model used for thermosetting materials.

**Fig. 20:**
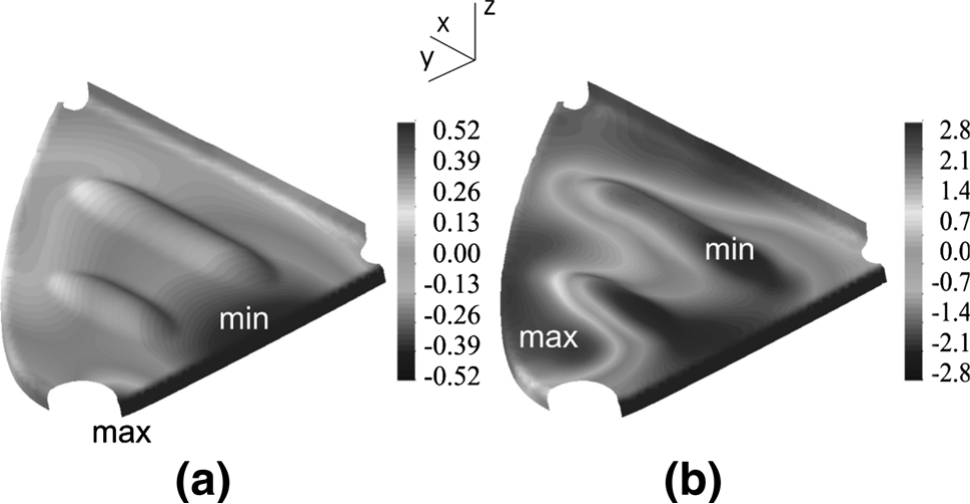
Deformed mesh of the wing leading edge rib after cooling, relative to the mesh after trimming. The *contours* indicate the out-of-plane displacements of the product in mm, with thermal stresses only (**a**) and with both thermal and fiber stresses taken into account (**b**) [183]

In Fig. 8, the degree of crystallinity (*X*), elastic modulus and linear shrinkage strain of a PEEK resin are plotted together with temperature change during the cooling ramp of the process. The degree of crystallinity reaches its maximum value at approximately 7 min after the cooling starts from the process temperature ($375\,{}^\circ \hbox {C}$) with a cooling rate of $2\,{}^\circ \hbox {C}$. The effect of crystallization is reflected as a small step change in development of the elastic modulus and shrinkage strain. A substantial increase of two orders of magnitude in the modulus is observed at the glass transition temperature which takes place approximately 15 min after the cooling starts. The PEEK changes its states from a rubbery solid to a glassy solid at the glass transition and the CTE also changes which can be seen in Fig. 8 as a change in the slope of the shrinkage strain plot.

#### Stress–Strain Relation

Process induced stresses and displacements are incrementally solved using the FEM. The total incremental strain ($\dot{\varepsilon }^{tot}$), which is composed of the incremental mechanical strain ($\dot{\varepsilon }^{mech}$), thermal strain ($\dot{\varepsilon }^{th}$) and chemical strain ($\dot{\varepsilon }^{c}$), is given in Eq. 39. Here, the incremental process induced strain ($\dot{\varepsilon }^{pr}$) is defined as the summation of $\dot{\varepsilon }^{th}$ and $\dot{\varepsilon }^{c}$ as also done in e.g. [34, 35, 58]. The incremental stress tensor ($\dot{\sigma }_{ij}$) is calculated using the material stiffness matrix ($C_{ijkl}$) which is a function of temperature and degree of polymerization based on the incremental mechanical strain tensor ($\dot{\varepsilon }_{ij}^{mech}$) (Eq. 40).

39 $$\begin{aligned} \displaystyle \dot{\varepsilon }_{ij}^{tot}= \, &{} \dot{\varepsilon }_{ij}^{mech} + \dot{\varepsilon }_{ij}^{th} + \dot{\varepsilon }_{ij}^{c} \nonumber \\ \displaystyle \dot{\varepsilon }_{ij}^{pr}= \, & {} \dot{\varepsilon }_{ij}^{th} + \dot{\varepsilon }_{ij}^{c} \nonumber \\ \displaystyle \dot{\varepsilon }_{ij}^{mech}= \, &{} \dot{\varepsilon }_{ij}^{tot} - \dot{\varepsilon }_{ij}^{pr} \end{aligned}$$

40 $$\begin{aligned} \dot{\sigma }_{ij}= \, & {} C_{ijkl} \dot{\varepsilon }_{kl}^{mech} \end{aligned}$$

Note that the fiber stresses should be taken into account for the constitutive modeling for thermoplastic composite manufacturing processes since the fiber stresses occur due to draping [38, 93]. The fiber stress can be treated as the elastic, thus non-relaxing in the fiber direction. It was shown by Wijskamp in [38] that the transverse shear loading of the laminate caused fiber stresses, which were subsequently frozen-in upon solidification and hence resulted in warpage formation in the manufactured part.

The stress and strain tensors are updated at the end of the each time increment *n* as in Eqs. 41 and 42, respectively [94].

41 $$\begin{aligned} \sigma _{ij}^{n+1}= \, & {} \sigma _{ij}^n + \dot{\sigma }_{ij}^n \end{aligned}$$

42 $$\begin{aligned} \varepsilon _{ij}^{n+1}= \, & {} \varepsilon _{ij}^n + \dot{\varepsilon }_{ij}^n \end{aligned}$$

### Viscoelastic Model

The viscoelastic constitutive models have been used to predict the residual stresses and shape distortion by taking the stress relaxations into account during the polymerization process. The viscoleastic behavior is inevitably present in the composite manufacturing processes especially when under elevated temperatures and long polymerization times such as in the RTM [64]. There are two different forms of viscoelastic models: differential and integral form. Generally, the integral form has been used in literature by several researchers [64, 95–103].

43 $$\begin{aligned} \sigma _{ij} = \int _0^t C_{ijkl}(\psi ,T,\xi -\xi ^\prime )\frac{\partial \varepsilon _{kl}(\xi ^\prime )}{\partial \xi ^\prime } d\xi ^\prime \end{aligned}$$

where

44 $$\begin{aligned} \xi (t)= \, &{} \int _0^t \frac{dt}{\chi (\psi ,T)} \end{aligned}$$

45 $$\begin{aligned} \xi ^\prime (t^\prime )= \, &{} \int _0^{t^\prime } \frac{dt^\prime }{\chi (\psi ,T)} \end{aligned}$$

and $\xi$ is the current reduced time, $\xi ^\prime$ is the past reduced time, *t* is the current time, $t^\prime$ is the past time, $\chi$ is the temperature (*T*) and degree of cure or crystallization ($\psi$) dependent shift factor. The relaxed modulus of the matrix material can be approximated using the Prony series expressed as [96]:

46 $$\begin{aligned} E_r(\psi ,T,t) = E_r^{rel} + \left( E_r^{rel} - E_r^{unrel}\right) \sum _i^n w_i \exp \left[ \frac{-\xi (\alpha ,T)}{\tau (\psi )} \right] \end{aligned}$$

where $E_r^{rel}$ is the fully relaxed modulus, $E_r^{unrel}$ is the unrelaxed modulus, $w_i$ is the weight fitting factor and $\tau (\psi )$ is the discrete relaxation times as a function of degree of polymerization.

The characterization of viscoelastic parameters needs dedicated experiments and they are in general more cumbersome to determine than the parameters used in the elastic model, e.g., the CHILE model. However, viscoleastic models predict the mechanical behaviour more accurately than the elastic models. A viscoelastic model was developed in [104] for the FM-94 epoxy resin using the Prony series given in Eq. 46. The viscoelasticity during curing of the FM-94 epoxy was investigated experimentally using the TMA and DMA together with DSC as was also done in [105]. On the other hand, a numerical algorithm was developed in [106] to characterize the viscoelastic properties of polymers using the vector fitting method which is commonly used for system identification in electronics and automated control. The fitted model, which switched the relaxation modulus from the frequency domain to the time domain, was validated with the experimental data for the viscoelastic properties of no-flow under fill materials. In [107], a cure dependent relaxation modulus was characterized for the resin system EPON Resin 862 based on the study presented in [96]. A rheometer was utilized in plate-plate mode to measure the material behavior below the gel point and creep tests were also conducted in three point bending conditions while above gelation. A viscoelastic model was fitted to the experimental data. It was found that the peak relaxation time showed a significant increase near the gel point. Moreover, the elastic response was found to be almost independent of curing state.

### Path Dependent Model

A simplified version of the viscolestic model was proposed in [12, 66, 67, 92] by introducing a path dependency instead of a rate dependency for the material bahaviour. The glass transition temperature was taken into account for the material stiffness matrix and the stress relations. The corresponding stress-strain relations can be expressed as:

47 $$\sigma _{ij} = \left\{ \begin{array}{lll} \displaystyle C_{ijkl}^r \varepsilon _{kl}, &{}\qquad T^* \le 0 \\ \displaystyle C_{ijkl}^g\varepsilon _{kl} - \left[ \left( C_{ijkl}^g - C_{ijkl}^r\right) \varepsilon _{kl} \right] _{t=t_{vit}}, &{}\qquad T^* > 0 \end{array}\right.$$

where $t_{vit}$ is the time of the last rubber-glass transition (vitrification) and the subscripts *r* and *g* state the rubbery and glassy, respectively [67]. The incremental formulation of Eq. 47 can be written as [67, 92]:

48 $$\dot{\sigma }_{ij} = \left\{ \begin{array}{lll} \displaystyle C_{ijkl}^r\dot{\varepsilon }_{kl} - S_{ij}(t), &{}\qquad T^* \le 0 \\ \displaystyle C_{ijkl}^g\dot{\varepsilon }_{kl}, &{}\qquad T^* > 0 \end{array}\right.$$

where $S_{ij}$ is a state variable accounting for the loading history particularly for stress relaxation. In the glassy region the state variable stores the stresses whereas it becomes zero in rubbery region. This situation can be mathematically expressed in the incremental form as [67, 92]:

49 $$\begin{aligned} S_{ij}(t+\Delta t) = \left\{ \begin{array}{lll} \displaystyle 0, &{} \qquad T^* \le 0 \\ \displaystyle S_{ij}(t) + (C_{ijkl}^g - C_{ijkl}^r)\dot{\varepsilon }_{kl}, &{}\qquad T^* > 0 \end{array}\right. \end{aligned}$$

The elastic modulus of the matrix material was modelled using a step change at the vitrification point ($t_{vit}$) as seen in Eq. 50. The rubbery properties were assumed to be about two orders of magnitude smaller than those in the glassy state.

50 $$\begin{aligned} E_r = \left\{ \begin{array}{lll} \displaystyle E_r^r, &{}\qquad T^* \le 0 \\ \displaystyle E_r^g, &{}\qquad T^* > 0 \end{array}\right. \end{aligned}$$

### Equilibrium Conditions

The variation of the stress components as functions of position within the interior of a body is determined in the stress analysis. This can be considered as a type of boundary value problem often encountered in the theory of differential equations, in which the gradients of the variables, rather than the explicit variables themselves, are specified. In the case of stress, the gradients are governed by conditions of static equilibrium. Let the surface traction at any point on a surface *S* be the force $\mathbf {t}$ per unit of area, and let the body force at any point within the volume of material (*V*) under consideration be $\mathbf {f}$ per unit of current volume. Then, the force equilibrium for this volume *V* is written as:

51 $$\begin{aligned} \int _S \mathbf {t} dS + \int _V \mathbf {f} dV = 0 \end{aligned}$$

The “true” or Cauchy stress matrix ${\varvec{\sigma }}$ at a point of *S* is defined by

52 $$\begin{aligned} \mathbf {t} = \mathbf n \cdot {\varvec{\sigma }} \end{aligned}$$

where $\mathbf {n}$ is the unit outward normal to *S* at the considered point. This yields in:

53 $$\begin{aligned} \int _S \mathbf n \cdot {\varvec{\sigma }} dS + \int _V {{\varvec{f}}} dV = 0 \end{aligned}$$

Using Gauss’s theorem, the surface integral can be rewritten as a volume integral. Hence, the equilibrium equation given in Eq. 53 is expressed as:

54 $$\begin{aligned} \int _S \mathbf n \cdot {\varvec{\sigma }} dS = \int _V \nabla {\varvec{\sigma }} dV = 0 \end{aligned}$$

Since the volume *V* is arbitrary, this requires that the integrand be zero:

55 $$\begin{aligned} \nabla {\varvec{\sigma }} = 0 \end{aligned}$$

Equation 55 ensures the equilibrium conditions based on the stress-strain relation defined in Eq. 40 for linear elastic, Eq. 43 for viscoelastic and Eq. 47 for path-dependent constitutive models in which the total strain is decomposed into its components as in Eq. 39. Hence, the static equilibrium in Eq. 55 can be written as a function of the material stiffness matrix and displacements through partial derivative equation system.

## Modelling the Tool–Part Interaction

Several numerical modelling approaches have been developed to capture the effect of tool–part interactions [15, 34, 58, 108–114]. In these models, the tool–part interaction was modelled as a cure hardening elastic shear layer which remains intact until the tool was removed [34, 58, 112], as an interfacial sliding friction at the tool-composite part interface [15, 114, 115], and as the part stuck to the tool surface with no relative motion [113]. These models are semi-empirical models that need to be calibrated with the use of experimental data. By adjusting the shear layer properties such as the elastic and shear moduli, the amount of stress transferred between the tool and part can be tailored and a range of tool-part interface conditions can be simulated. According to a parametric study [116] using the shear layer model, the tool-part interfacial shear stress distribution is critical for accurate modelling of distortions. Arafath et al. [110, 111] also used this shear layer assumption in a closed-form solution for process-induced stresses and deformations for flat and curved geometries. A parametric study examined the effects of the elastic and shear moduli and thickness of the shear layer on the compaction behaviour, and concluded that improving the ability of the flexible male mould to slip against the laminate could be helpful to apply pressure to the L-shaped laminate at the corner side [117]. In some studies [15, 32, 108, 114], the interfacial shear stress was assumed to obey the Coulomb friction model [118]. This models the interfacial shear stress as being proportional to the contact pressure, where the constant of proportionality is the fiction coefficient, up to a critical shear stress beyond which sliding with a constant shear stress is observed.

The effect of the tool–part interaction on the distortion has been emphasized in recent studies [34, 58, 59, 68, 110–117, 119–130]. Twigg et al. [116, 119, 120] conducted experimental and numerical studies to understand the mechanics and constitutive behavior of the tool–part interface. To examine the interaction between the tool and part, an instrumented tool method was introduced [119]. The critical interfacial shear stress ($\tau _{cr}$) at which sliding occurs was modelled using the following expression [119]:

56 $$\begin{aligned} \tau _{cr} = \mu \cdot P \end{aligned}$$

where $\mu$ is the coefficient of friction and *P* is the pressure acting normal to the tool-composite part interface. The stick-slip behaviour was determined based on $\tau _{cr}$ which was defined as a degree of cure as illustrated in Fig. 9. It is seen that the shear stress at the interface increases with an increase in degree of cure. A strain gage mounted on a thin aluminium sheet was laid on the flat carbon/epoxy laminate to measure the strains imposed on the aluminium sheet by the tool–part interactions as a function of time during the cure cycle. It was concluded that a sliding friction condition occurs during the heat-up portion of the cure cycle, and that the value of the sliding shear stress increases with the degree of cure. During temperature modulations and cooling, a sticking interface condition dominates the interaction. Also it was shown that the use of a FEP release film prevents the sticking of the laminate to the tool, but that using a release agent causes adhesive bonding at the interface. In an experimental study of the same group, the effect of part aspect ratio and processing conditions on warpage were investigated [120]. For a given lay-up and material, the part aspect ratio was found to be more effective than pressure at reducing warpage, while the magnitude of warpage was not influenced significantly by the tool surface condition.

As opposed to the study conducted in [120] in which the strain gage was embedded to the tool, a strain gage was directly mounted on the prepreg by using the spot curing technique [15, 119], where a small spot of uncured prepreg was cured to provide a bonding location for the strain gage. After mounting the strain gage on the prepreg and before moulding, the strain gage was calibrated using a tensile test machine to determine the modulus of the prepreg. The prepreg was then cured using the MRCC, and the strain was recorded as a function of time. Fiber Bragg Grating (FBG) sensors (optical fiber sensors) were embedded along the reinforcement orientations in [126, 128, 129] to measure the strains developing due to the expansion of the tool during curing. The residual strains were examined in [126] using four different mould materials, i.e., aluminium, steel, carbon foam and carbon/epoxy composite based. Higher strains were measured in the composite part using the aluminium and steel moulds than the strains for carbon foam and carbon/epoxy moulds. It should be kept in mind that the FBG sensors can only measure the strains after gelation since the mechanical interlocking between the sensor and the resin is only possible after gelation, whereas the instrumented tool and instrumented ply techniques can measure the tool–part interaction stresses in the early stages of curing when the resin is in the viscous state. Experimental setups were designed to quantify the friction coefficient and shear stress at the tool-part interface [68, 114, 124, 125, 130] in which two temperature controlled heated platens and a loading plate were utilized. The prepreg was then pulled against other prepreg layers or tool material in a tensile testing machine. After the heated platens were pushed towards each other using a pneumatic ram [124, 125] or a spring screw set [68] to simulate autoclave pressure, the tensile test machine applied a load and recorded the load vs. displacement. Ersoy et al. [68] measured the interfacial shear stresses between the tool and the part directly in terms of temperature and pressure. They observed significant tool–part interaction stresses, presumably due to fiber friction that developed during the early stages of curing, even when the resin was in viscous state.

Friction coefficients during the curing were measured in other studies [114, 124, 130]. Martin et al. [130] and Flanagan et al. [114] measured only the static friction coefficient during the ramp-up portion of the cure cycle. Their observations [68, 130] showed that the tool-part interface changes during the process cycle. In [125], both static and dynamic friction coefficients were measured as a function of the degree of cure, ramp rate and pressure for the entire cure cycle [124, 125]. A non-linear relationship was observed between the friction coefficient (static and dynamic) and the degree of cure. This indicates that the mechanical interface condition changes from a sliding to a sticking condition as the cure advances. This is also confirmed by the observation that there is less resin residue at the interface as the cure advances indicating a shift from cohesive to adhesive failure. Regarding the roughness and anisotropic expansion of the tooling material, it was found in [122, 123] that a variation in the surface roughness affected the warpage formation, though the anisotropic expansion of the raw tooling material from the rolling process did not cause any significant change. The shear stress transfer from the tool to the parts was also examined using different surface conditions and prepreg systems. One result of the study was that the M21/T800 and AS4/8552 prepreg systems showed considerable deformation when using a release film. By contrast, when the same release film was used for the 977-2/IM7 prepreg system, the deflection was found to be considerably smaller.

A friction model was developed in [131] to predict the friction between a glass fiber reinforced polypropylene (Twintex) laminate and a rigid tool during melting conditions. The friction model was based on the hydrodynamic lubrication theory in meso-mechanical scale. Reynolds equation was used in the friction model for thin film lubrication and only the rheological properties of the matrix material and the fabric weave geometry were utilized as input parameters. In [132], a comparison between different friction test set-ups for thermoplastic composites was made in a friction benchmark exercise. Schematic representation of the friction test set-ups of the benchmark participants is depicted in Fig. 10. The material considered in the experiment was the Twintex polypropylene material. Two different types of friction were tested: *i)* dry friction at ambient temperature which was an Amontons-Coulomb-like friction and *ii)* hydrodynamic friction at temperatures above the melting point of the resin. It was concluded that larger friction surfaces reduced the influence of edge effects and chamfered edges or pulling the metal foil instead of the composite material can minimize these edge effects. Friction experiments were performed in [133] on a capstan and a plate friction type setup. The measurement results were found to quantitatively be comparable, although the setups relied on two different load application methods. The agreement of the results formed a validation of both friction characterization methods.

## Applications to Thermosetting Composites

In the extensive published literature, researchers have spent considerable effort to predict residual stresses and distortions in fiber reinforced composites using analytical and numerical methods. An early example of the analysis of the residual stress generation using the laminated plate theory considered only the cool down stage of the process and was only in 1D [16]. Different contributions from material anisotropy, cure shrinkage, tool–part interaction and resin flow have a significant effect on residual stresses and shape distortions as aforementioned. Some of these mechanisms occur from the beginning to the end of the manufacturing process or occur only at some stages of the process. In order to address this complexity, more sophisticated process models have been developed in literature [34, 35, 75, 134]. In the following sections the process models are grouped according to their manufacturing methods such as autoclave curing, RTM, pultrusion, and vacuum infusion. By doing so, one gets the opportunity to compare the characteristics of the process models with the specific evolution of the residual stresses and deformations.

### Autoclave Curing Method

The CLT was used in the early studies which simulated the autoclave curing process of continuous fiber reinforced composite materials [35, 36, 134–136]. Loos and Springer [135] developed a process model for the curing of flat-plate composites. The process model provided the temperature distribution, the degree of cure of the resin, the resin viscosity inside the composite, the void size and the residual stress distribution after the cure. An integrated sub-model approach was introduced to divide this complex problem into simpler sub-models. The integrated sub-model approach was also used by Bogetti and Gillespie [35, 36] and by White and Hahn [75, 134] to predict process induced stresses and deformations. Bogetti and Gillespie [35] developed a 1D curing simulation coupled to the CLT for thick composites which included temperature gradients through the thickness, spatially varying cure dependent mechanical properties, thermal expansion and chemical shrinkage strains.

Radford [24] investigated the effect of fiber volume fraction variations on the residual curvature formation using CLT. The mid-plane curvatures were determined using the CTE of the composite part and shrinkage strains of the matrix material. The observed curvature behavior was more pronounced in thin laminates, however the measurable warpage did exist even in thicker composites. The results of these preliminary quantitative observations indicated that many laminates were measurably resin-rich at the composite/tool interface and resin-poor at the laminate top surface where bleeding took place.

White and Hahn [75, 134] developed a process model which predicted the transient stress history during the curing of composite materials by including the effects of chemical and thermal strains. The relation between the degree of cure and mechanical properties of the composite was modelled using a power law expression. CLT was used for the calculation of the elastic residual stresses and the quasi elastic method was used for the calculation of the viscoelastic residual stresses. The cure kinetics model was combined with the viscoelastic stress analysis to estimate the residual bending moment and curvatures simultaneously for unsymmetrical cross-ply flat laminates and the tool–part interaction was not included.

Arafat et al. [110, 111] developed a closed-form solution using elasticity theory for the prediction of the process induced stresses and deformations in flat and curved composite structures. A 2D analytic process model was combined with the CHILE constitutive model thus resembling the classical bi-metallic beam under a thermal load and the axial stress distribution in the through-thickness directions depended on the material properties which in turn changed during the cure. The stress gradients were controlled by the ratio of the elastic modulus in the fiber direction to the transverse shear modulus. It was concluded that the material properties at early stages of the curing process led the final mechanical response of the part. It should be noted that the CLT based models are practically restricted to use simplified mechanical boundary conditions, hence relatively complex shaped composite parts are difficult to model with the CLT.

For complex shaped parts, FE analysis has been widely utilized to investigate the process induced stresses and deformations in composite forming processes [58, 83, 108, 109, 113, 129, 137–141]. Johnston et al. [58] developed a 2D plane strain FE model which employed the CHILE constitutive model. The effect of the CTE, cure shrinkage, temperature gradients, degree of cure, resin flow and mechanical constraints on the deformation of the composite laminates was investigated using an integrated sub-model approach. The tool–part interaction was modelled by an elastic “shear layer” that remained active until the tool was removed [58]. Their predicted spring-in values were found to agreed with the measured spring-in angle for a $[0]_{24}$ lay-up, however a deviation was found between the calculated and measured spring-in angle for $[90]_{24}$ composite laminates.

Ersoy et al. [83] developed a two-step 2D FE model to predict the process induced stresses and deformations in composites parts having an anisotropy in the CTE and cure shrinkage. The two-step model represented the rubbery and glassy states of the resin. The reason for preferring a two-step approach was to overcome the complexity of specifying the continually changing material properties during the cure cycle. Constant material properties were used in each step. The gelation took place at approximately 30 % degree of cure and vitrification occurred at approximately 70 % degree of cure for the resin. Gelation and vitrification were considered to be the two main transitions during the curing process and utilized in the two-step model. Rubbery material properties were used in the first step of the model before vitrification, whereas glassy material properties were used in the second step after vitrification. The properties of the composite in the glassy state were determined both experimentally and numerically. The predictions of the glassy properties were found to be very close to the experimental values [44]. In this study, a part geometry and tool material were chosen to minimize the issues related to tool–part interaction and consolidation. Hence, the stresses that developed before gelation were ignored. Spring-in values predicted by the two-step FE analysis were very close to corresponding measurements for both unidirectional and cross-ply C-shaped composite parts. The predictions followed a trend of decreasing spring-in with increasing thickness, which matched the experimental results well. The measured spring-in angles were also very close to the predicted ones for the thicker parts, and slightly underestimated for the thinner parts with a maximum difference of 15 %. In [82], the phenomenon of decreasing spring-in with increasing thickness was explained by the shear deformation (shear-lag) between plies. The aim was to maintain the same arc length during curing while decreasing the amount of in-plane stress which caused smaller spring-in values [82].

Zhu et al. [113] developed a 3D coupled thermo-chemo-viscoelastic model to simulate the heat transfer, curing, residual stresses and deformation of thin flat and L-shaped composite laminates. It was found that a thermo-chemo-viscoelastic model gave much larger spring-forward values as compared to a elastic or viscoelastic model that only accounts for the cool-down process.

A design tool was developed by Cilfford et al. [138] to predict the residual stress and dimensional stability of large complex shaped composite parts. The spring-in values of asymmetric cross-ply V-shaped laminates made of non-crimp carbon fabric reinforced epoxy were predicted using a 3D FE model using an anisotropic thermo-viscoelastic formulation. A hybrid finite element mesh, containing both shell and solid elements, was proposed to minimize the computational time. It was concluded that the approach was valid; however, the numerical model over-predicted the deformations as compared to experimentally observed part deformations [138].

Cinar et al. [108] extended the finite element procedure developed by Ersoy et al. [83] to include tool–part interaction to predict the manufacturing distortions of corner sections, as shown in Fig. 11. The effects of various material and geometric variables on the deformation of L-shaped composite parts were investigated using a parameter sensitivity analysis [108]. The effect of the sliding shear stress and viscous shear modulus on spring-in predictions for UD-R25 parts is given in Fig. 12. Figure 13 shows the numerical results for the through-thickness residual stresses in the fiber direction obtained from the developed FE model for the process simulation of the UD4-R15 part. The stresses were calculated according to the local coordinate system shown in Fig. 11. It can be seen that the stress in the fiber direction, $\sigma _{22}$, increases towards the corner of the part. It has been observed that tensile stresses occur at the corner for both bag and tool side during the 1st and 2nd steps. Consolidation and cure shrinkage taking place during the 1st and 2nd steps caused stretching of the fibers on the bag side. and the tool–part interaction caused stretching of the fibers on the tool side [109]. The resulting stress distribution was in tension in the through-thickness direction and relatively higher tensile stresses were present at the tool and bag side as compared with the center of the part. Cinar [109] also performed a 3D FE analysis of long L- and U-shaped composite parts using a 3 step model. The predictions matched the manufactured counter parts very well. Measured and predicted deformation patterns for $[0]_4$ and $[0/90]_s$ L-shaped parts with a 15 mm corner radius are given in Fig. 14. In [32], an initial conformation step was added to the FE based approach to simulate the initial compressive strains and to capture the fiber wrinkling at the corner sections during the lay-up process of prepregs. It was shown that the fiber wrinkling decreased the spring-in angle by reducing the fiber tension in the inner fibers of the curved part.

An integrated sub-model approach was used in [139] similar to the study conducted in [35]. A 3D coupled thermomechanical FE model was developed taking the cure kinetics, cure shrinkage, thermal strains, tool–part interaction and the development of mechanical properties during curing into account. A DMA was used to determine mechanical properties in different directions. Two types of interactions were considered for the tool–part interface: (*i*) a mechanical interaction dealing with the frictional forces, (*ii*) a thermal contact for heat transfer. The Coulomb friction model with stick-slip behavior was used for the mechanical interaction. A square flat laminate was manufactured to validate the simulations. The predictions and experimental results for maximum distortion and the deformation pattern were found to be in good agreement.

The optimization of a tooling geometry was performed in [140] to compensate for the cure-induced deformations. A doubly curved C-spar geometry was optimized to minimize process-induced deformations using the control points method in which the residual distance was measured between the current position of the control points and their nominal position after a best-fit alignment. However, the approach was not validated experimentally.

Some other approaches were also used to predict process-induced distortions [122, 142] in composite manufacturing processes. A piece-wise approach was introduced in [142] to calculate the dimensional changes in curved composite parts by dividing the curved structure into a number of pieces and calculating deformations using the effective CTE. The approach was validated using a FE model and satisfactory results were obtained. A semi-numerical methodology was used in [122] in which the experimental, analytical and numerical approaches were combined in order to predict the cure-induced shape distortions. The residual spring-in values were measured for the L-shaped composite parts and subsequently utilized in the FE model to calculate the deformations of more complex parts, e.g., a box-shaped structure.

In [143], the process induced residual stresses and shape distortions were predicted using a viscoelastic model during the curing of a graphite/epoxy composite system. A general purpose FE software ABAQUS was employed for the model development. A carbon foam tooling was employed and it was shown that the residual stress level reduced with the carbon foam.

A 3D thermo-viscoelastic model was developed in [138] to predict the deformations in the V-shaped parts manufactured by autoclaving. Asymmetric cross-ply laminates made of non-crimp carbon/epoxy were considered. It was found that the model over-predicted the deformation magnitude and the effect of the tool–part interaction was found to play a relatively small role. The residual stresses were calculated analytically in [144] using a linear viscoelastic model. FBG sensors were utilized to measure the evolution of the strain. Significant stress gradients were found in [145] using a 3D incremental viscoelastic constitutive equation developed in ABAQUS. An integrated T-shaped composite structure was considered in [145] which was produced using the autoclaving. The residual stresses were analyzed in [146] computationally and correlated with the mechanical properties of the carbon/epoxy laminates produced by autoclaving. The effects of the curing process on the residual tresses as well as on the mechanical performance were investigated using three different curing cycle. Measurement of the out-of-plane shape distortions showed that different curing conditions were able to generate different residual stress levels. It was also shown that the residual stresses were beneficial for the transverse tensile strength of unidirectional (UD) lamiantes.

The effects of autoclave curing pressure and cooling rate on the residual shape distortions were studied in [147] for [$0_2/90_2$] carbon/epoxy laminated strips. It was found that changing the cooling rates had no significant influence on the residual shape distortions, on the other hand the autoclave pressure played an important role.

### The RTM Method

In the manufacturing of complex shaped composite parts, the RTM is one of the widely used techniques which involves transfer of resin into a closed mold containing previously laid up dry reinforcement preforms. RTM simulations have been carried out in many studies to predict residual stress and deformations [37, 59, 66, 67, 95, 148–151]. Process models were generally divided into sub-models including heat transfer and cure kinetics, flow and compaction and stress development [37, 59, 66, 67, 149].

Using an incremental approach [37, 59, 148, 149],cure-dependent elastic models were used to compute the resin elastic modulus during the entire cure in which the resin elastic modulus was linearly dependent on the degree of cure [148, 149]. A temperature- and cure-dependent resin elastic modulus was developed in [59].

Cure-dependent mechanical properties were employed in [149] using a 3D FE based model developed for the RTM method to evaluate the process-induced residual stresses and deformations. The model investigated the effect of thermal strains and cure shrinkage on the stresses and deformations. A perfect mechanical bonding was used at the tool-part interface. The nodes along the interaction region were released at the cool down stage in which the resin shrinkage was neglected. The residual stresses and distortion were predicted for a tapered composite structure.

A 2D semi-numerical model was developed for the process modeling of a relatively thick woven fabric composite plate [148]. The incrementally employed model consisted of resin cure kinetics and volumetric shrinkage. The numerical approach used a cure dependent woven fabric unit cell model and a FE structural analysis. The chemical hardening of the resin modulus and the volumetric cure shrinkage of the resin was assumed to depend linearly on the degree of cure in the model. Process induced stress and strains were predicted, however not verified by comparison with experimental data. The unsymmetrical curing strain which was the highest at the middle and the lowest at the bottom of the plate resulted in a concave-shaped warpage.

Svanberg and Holmberg [12, 66, 67] developed a simplified mechanical constitutive model to predict the shape distortions of an L-shaped bracket and a C-spar structure. As opposed to the incremental approach they assumed that the mechanical behavior of the material was constant within the rubbery and glassy states [67] and there was a step change in the properties at the glass transition temperature as explained in Sect. 4.3. Three different tool–part interaction models were used in the developed FE analyses: freestanding, fully constrained and frictionless contact conditions. The model indicated that the contact boundary conditions gave the closest agreement to the measured spring-in. There was no experimental data to justify the numerical findings in the rubbery state and the tool–part interaction was oversimplified. A comparison between the predicted and experimental shape deformations indicated that the predicted spring-in showed good agreement with the experimental values after the second cure step, however the predicted values deviated after the third cure step, i.e., the predicted spring-in angle was overestimated after the third cure step.

Mechanical contact constraints were well implemented in a 3D FE model in [59, 151] to take the tool–part interaction into account. Three different mechanical contact interactions between the tool and the laminate were investigated: no bonding, perfect bonding and frictional contact. Frictional contact consisted of a stick-slip behavior based on the Coulomb friction. The frictional contact interaction model addressed the evolution of the strains measured by FBG sensors, embedded in the composite laminate.

The residual stress development was captured in [95] by implementing a viscoelastic model using the classical laminate theory (CLT). The thermal mismatch during the cooldown stage was found to be the dominant effect for the residual stress build up since the aluminum mold applied a strong constraint effect on the strain built up. The viscoelastic modulus was obtained by conducting DMA experiments for stress relaxation. The curvatures of $[0^\circ /90^\circ ]$ asymmetric laminates were calculated and compared with corresponding curvature measurements.

Dong et al. [150] developed a regression-based dimensional variation model to study the angled structure and single/multiple stiffener structures using a FE based model. The approach enabled to compute the deformation of composite assemblies by the introduced structural tree method. The advantage of the approach was to reduce the computation time for the industrial applications.

### The Pultrusion Process

A state-of-the-art numerical modelling framework was developed by Baran et al. [152] to predict the residual stresses and shape distortions in pultrusion processes. An FE model was employed in which a 3D thermo-chemical model was coupled with a 2D quasi-static mechanical model. A schematic view of the sequential coupling can be seen in Fig. 15. A Eulerian frame of calculation was considered in the 3D thermo-chemical analysis in which the material flowed within a stationary mesh. On the other hand, the 2D cross section of the composite was assumed to advance through the pulling direction for the 2D mechanical analysis. This can be considered a Lagrangian frame in which the mesh is translated with the corresponding time frame. A more advanced mechanical model was developed in [153] using full 3D elements instead of 2D plane strain elements. In this case the 3D stress state evolution was predicted using the proposed approach given in [153] for UD square profiles made of glass/epoxy. The evolution of the transient stresses and distortions was captured and the obtained results were compared with each other using the three different mechanical models: 2D plane strain, 2D generalized plane strain and 3D models.

A UD graphite/epoxy rod was studied in [154] in which the diameter of the rod was 9.5 mm. The stress levels were found to be relatively small ($<$1 MPa) due to the quite uniform temperature and curing evolutions in the part. Therefore, there is a relatively small through thickness temperature and cure gradient present in the product which does not promote the generation of stresses.

The NACA0018 airfoil profile was considered in [155–157]. The residual stresses were predicted for the pultrusion process of the blade profile in [157]. It was found that the transient normal stresses in the pulling direction were higher than the normal stresses in the transverse direction inside the die. However, this turned out to be the other way around at the end of the process. The residual stresses and the final mechanical properties of the NACA0018 profile calculated in the pultrusion process simulation were transferred to the subsequent bending simulation. The residual stresses were treated as a pre-stress condition before the bending simulation. Subsequently, a loading scenario was carried out and the internal stress levels of the bent profile were evaluated.

A thermo-chemical-mechanical analysis of the pultrusion process was presented in [158]. A process simulation was performed for an industrially pultruded rectangular hollow profile $(64\times 27\times 3\hbox { mm})$ containing both unidirectional (UD) roving and continuous filament mat (CFM) layers. The reinforcements were impregnated with a commercial polyester resin mixture (Atlac 382). Figure 16 shows the predicted warpage behavior which was found to match well the warpage observed in the real pultruded rectangular hollow products seen in Fig. 16.

A numerical simulation tool was developed to calculate the process induced stresses and dimensional variations in an industrially pultruded L-shaped profile in [159]. The cross sectional dimensions of the part were $50\times 50\times 5\hbox { mm}$ and it contained glass/polyester based UD roving and CFM layers. An “orthophthalic” polyester resin system was utilized to impregnate the reinforcements. Figure 17 shows the pultruded part together with the predicted residual spring-in angle ($\theta$) and the maximum principle stress ($S_{\max }$) distribution. The CLT [160] was employed to verify the predicted through-thickness residual stress field.

### Vacuum Infusion Method

In [64], the residual stresses and shape distortions were predicted using a linear elastic approach [34], a viscoelastic approach [96] and a path dependent method [92] for vacuum infusion processes of thermosetting composites. The process induced deformations in a commercial wind turbine blade root subsection were analyzed using the CHILE constitutive approach. It was found that the large non-uniform through-thickness temperature and degree of cure gradients play a significant role for the development of process induced shape distortions.

An L-shaped composite structure was modeled using a 3D thermal-elastic steady state FE analysis in [81]. Thermal contraction strains and polymerization shrinkage strains were taken into consideration and directly implemented to the model. The peak temperature and the room temperature were both experimentally measured in the study so that the transient heat transfer and cure simulation were not considered in the FE modeling.

The accuracy of the CHILE constitutive model was evaluated in [161] for a simulation of the slow curing process-induced transverse strain development of a thick laminate plate. The *insitu* strains were measured suing FBGs during the vacuum infusion process. It was found that the CHILE model overestimated the residual strains when compared with the experimental results which was owing to the time-dependent stress relaxation of the thermosetting system.

## Applications to Thermoplastic Composites

The earliest process models for thermoplastic composites simulated the processing of parts in an autoclave or hot press [162, 163] in which heat transfer and crystallization were modelled. Thermoplastic tape laying or filament winding processes were of particular interest in terms of process simulation. Early studies include only transient heat transfer [164–168]. Since these processes were fast and complex, process simulation was thought to bring an insight on process optimization and reduce the trial and error iterations for obtaining parts that have strengths comparable to those manufactured in an autoclave. Since bonding, polymer degradation and void growth are the main concerns in terms of strength, these are the phenomena that have been of primary focus after transient heat transfer [169–172]. A 1D heat transfer analysis was combined with a crystallization kinetics model in [84, 173]. The main aim was to calculate residual stresses and determine the through-thickness temperature and crystallinity distributions. The incremental total process induced strain, which was the sum of the thermal strains and the crystallization shrinkage strain, was predicted. Finally, incremental residual stresses were calculated using the CLT. Total stresses were obtained by summing up the incremental values. A modified form of the SLS model was implemented in [84] to predict the process induced macroscopic in-plane strains as a function of temperature and the degree of crystallinity. A semi crystalline PEEK reinforced with carbon fiber (APC-2) was considered. Both neat and composite processing strains were evaluated. It was also found that the strains due to the crystallization, which was related to the cooling rate, were much smaller as compared with the strains from thermal contraction of the PEEK matrix. More advanced models taking the most of the physical phenomena into account for predicting the residual stresses and strains as well as shape distortions of composite parts were developed for autoclave/hot press moulding [19, 174, 175] and thermoplastic tape placement process [176, 177]. Process models were also employed to predict the residual spring-in of L-shaped sections made of thermoplastic composites by compression moulding [178–181] or roll forming[182]. In [180], the dimensional stability of a V-shaped composite made of polyamide-12/carbon fiber (PA12/CF) commingled yarn was studied. A thermo-viscoelastic model was implemented to predict the stress development together with the shape distortions. Different cooling rates were used during the manufacturing of parts and it was shown that the predicted residual stress level was approximately the half of the transverse tensile strength of the cross ply part.

A rubber press forming simulation was performed using the FE method in [183] for an Airbus A-380 wing leading edge stiffener, manufactured by Stork Fokker AESP. A schematic view of the part and the modelling approach are depicted in Fig. 18. The pre-form of the rib was modelled using an unstructured mesh of 11,055 elements. The size of the product is approximately $280\times 250\times 60\hbox { mm}$. A three-step method was utilized to model the shape distortions of the composite wing leading edge stiffener which was made of woven fabric (8H satin glass) reinforced polyphenylenesulphide (PPS) with a $[45^{\circ}/-45^\circ/0^\circ/90^\circ$]s lay-up. The predicted wrinkles after the draping simulation were found to agree with the observed wrinkles from the experiments. These wrinkles are indicated in Fig. 19 with arrows and occur at the nose and next to the nose of the rib. The predicted out-of-plane displacements due to the thermal stresses and fiber stresses after trimming are shown in Fig. 20. It was found that the shape distortions due to cooling were quite small when neglecting the fiber stresses.

In [184], the formability of two different composite materials used in aerospace industry was investigated. The materials were a quasi-isotropic laminates of PEEK with a UD carbon fiber reinforcement (UD/PEEK) and a woven glass fiber reinforcement PPS (8HS/PPS). Forming simulations were carried out using a commercial FE software package ANIFORM [185]. The predicted intra-ply shearing patterns were found to match with the experimental results. THe developed model predicted also the wrinkle formations.

## Conclusions and Future Trends

Processing of fiber reinforced polymer composites inherently involves multi-physical phenomena such as resin flow, state changes, heat transfer and solid mechanics. The diverse interrelation between the governing physics makes the process challenging and difficult to control. The presence of residual stresses is inevitable in composite manufacturing processes which might cause process induced defects such as cracking and delamination in the part. When there is a nonuniform residual stress distribution, residual shape distortions are more likely to build up which is not desired since they alter the dimensional accuracy.

**Table 1:** Studies carried out in literature for predicting the residual stresses and shape deformations as grouped with respect to the manufacturing process and utilized constitutive material model

	Linear elastic model		Path dependent model		Viscoelastic model	
	Stress calculation	Distortion calculation	Stress calculation	Distortion calculation	Stress calculation	Distortion calculation
RTM	[59, 148, 149]	[59, 148, 149]	[66, 67]	[66, 67]	[95]	[95]
Vacuum infusion	[64, 81, 161]	[64, 81, 161]	[64, 161]	[64, 161]	[64, 161]	[64, 161]
Pultrusion	[152–155, 157–159]	[152–155, 157–159]	–	–	–	–
Autoclave	[35, 129, 135]	[34, 58, 83, 108, 141]	–	–	[78, 134]	[113, 137, 138]

In this paper, the main mechanisms generating residual stresses as well as distortions in manufactured composite parts were reviewed. The necessary modelling approaches containing multi-physical phenomena such as thermokinetics, chemorheology, resin flow with the focus on thermomechanical analyses of the manufacturing processes were presented for thermoset and thermoplastic composites. A general overview was provided for the three different constitutive material models developed in literature: (*i*) linear elastic model [34, 35], (*ii*) viscoelastic model [96] and (*iii*) path dependent model [92]. The linear elastic and path-dependent approaches were found to be more favorable than the viscolelastic approach due to reasonable model accuracy, ease of implementation and use of relatively simple material characterization [64]. The modified CHILE model is a very elegant, yet simple way of taking the highly non-linear material behavior into account [57].

**Table 2:** Overview of the main mechanisms generating the residual stresses and shape deformations utilized in literature

	Thermo-mechanical anisotropy	Polymeri-zation shrinkage	Tool–part interaction	Resin flow and compaction	Fiber wrinkling	Temperature gradients
RTM	[37, 59, 66, 67, 80, 148, 149]	[37, 59, 66, 67, 80, 148, 149]	[59, 66, 67, 149]	–	–	[37, 80]
Vacuum infusion	[64, 161]	[64, 161]	–	–	–	[64, 161]
Pultrusion	[152–155, 157–159]	[152–155, 157–159]	–	–	–	[152–155, 157–159]
Autoclave	[34, 58, 83, 108, 134, 141]	[34, 58, 83, 108, 134, 141]	[34, 58, 108, 113, 114, 116, 138]	[34, 58]	[32]	[35, 36]

The studies carried out in literature focusing on the modelling of residual stresses and shape deformations occurring during the manufacturing processes were reviewed in detail. It was found that a higher amount of case studies have been applied to thermosetting composites than to thermoplastic composites. Therefore, in the present work, the process modelling studies were grouped based on the utilized constitutive models and specific manufacturing techniques. The corresponding summary is provided in Table 1. Moreover, the overview of the main mechanisms considered in modelling the residual stresses and shape distortions are shown in Table 2.

The process modelling efforts for predicting the residual stresses and global deformation patterns in composite manufacturing has reached a certain maturity. The research efforts in the future are probably going to focus on the micromechanical or mesomechanical defect formation during the process, e.g., fiber wrinkling [184, 186], microbuckling of fibers, void formation, as well as global layer buckling and resin percolation at corners. Subsequently, the effects of process induced defects on the mechanical performance is of utmost importance with the final aim of predicting the mechanical failure behavior of the composite part in service life.

## References

[1] Paulsen US, Madsen HA, Hattel JH, Baran I, Nielsen PH (2013). Design optimization of a 5 MW floating offshore vertical-axis wind turbine. Energy Procedia.

[2] Paulsen US, Madsen HA, Kragh KA, Nielsen PH, Baran I, Hattel JH, Ritchie E, Leban K, Svendsend H, Berthelsene PA (2014). DeepWind-from idea to 5MW concept. Energy Procedia.

[3] Laurenzi S, Marchetti M (2012) Advanced composite materials by resin transfer molding for aerospace applications, composites and their properties. Ning Hu (ed). ISBN: 978-953-51-0711-8. InTech. doi:10.5772/48172

[4] Wisnom MR, Gigliotti M, Ersoy N, Campbell M, Potter KD (2006). Mechanisms generating residual stresses and distortion during manufacture of polymer-matrix composite structures. Compos A.

[5] Parlevliet PP, Bersee HEN, Beukers A (2006). Residual stresses in thermoplastic composites—a study of the literature—part I: formation of residual stresses. Compos A Appl Sci Manuf.

[6] Parlevliet PP, Bersee HEN, Beukers A (2007). Residual stresses in thermoplastic composites—a study of the literature—part II: experimental techniques. Compos A Appl Sci Manuf.

[7] Parlevliet PP, Bersee HEN, Beukers A (2007). Residual stresses in thermoplastic composites—a study of the literature—part III: effect of thermal residual stresses. Compos A Appl Sci Manuf.

[8] Ito Y, Obo T, Minakuchi S, Takeda N (2015). Cure strain in thick CFRP laminate: optical-fiber-based distributed measurement and numerical simulation. Adv Compos Mater.

[9] Nelson RH, Cairns DS (1989) Prediction of dimensional changes in composite laminates during cure. 34th International SAMPE symposium and exhibition, vol 34, pp 2397–2410

[10] Sarrazin H, Kim B, Ahn SH, Springer GS (1995). Effects of processing temperature and lay-up on springback. J Compos Mater.

[11] Gigliotti M, Wisnom MR, Potter D (2003). Development of curvature during the cure of AS4/8552 [0/90] unsymmetric composite plates. Compos Sci Technol.

[12] Svanberg JM, Holmberg JA (2001). An experimental investigation on mechanisms for manufacturing induced shape distortions in homogeneous and balanced laminates. Compos A.

[13] Ersoy N, Potter K, Wisnom MR, Clegg MJ (2005). Development of spring-in angle during cure of a thermosetting composite. Compos A.

[14] Radford DW, Rennick TS (2000). Separating sources of manufacturing distortion in laminated composites. J Reinf Plast Compos.

[15] Garstka T (2005) Separation of process induced distortions in curved composite laminates. Ph.D. thesis, University of Bristol

[16] Hahn HT, Pagano NJ (1975). Curing stresses in composite laminates. J Compos Mater.

[17] Weitsman Y (1979). Residual thermal stresses due to cool-down of epoxy-resin composites. J Appl Mech.

[18] Adolf D, Martin JE (1996). Calculation of stresses in crosslinking polymers. J Compos Mater.

[19] Sunderland P, Yu W, Manson JA (2001). A thermoviscoelstic analysis of process-induced internal stresses in thermoplastic matrix composites. Polym Compos.

[20] Kevin D, Beaumont PWR (1997). The measurement and prediction of residual stresses in carbon-fibre/polymer composites. Compos Sci Technol.

[21] Hull D (1981). An introduction to composite materials.

[22] Radford DW, Diendorf RJ (1993). Shape instabilities in composites resulting from laminate anisotropy. J Reinf Plast Compos.

[23] Hubert P, Poursartip A (1998). A review of flow and compaction modelling relevant to thermoset matrix laminate processing. J Compos Mater.

[24] Radford DW (1995). Volume fraction gradient induced warpage in curved composite plates. Compos Eng.

[25] DA Darrow JR, Smith LV (2001). Isolating components of processing induced warpage in laminated composites. J Compos Mater.

[26] Lightfoot SC, Wisnom MR, Potter K (2013). A new mechanism for the formation of ply wrinkles due to sheer between plies. Compos A.

[27] Potter K, Khan B, Wisnom MR, Bell T, Stevens J (2008). Variability, fibre waviness and misalignment in the determination of the properties of composite materials and structures. Compos A.

[28] Bloom LD, Wang J, Potter KD (2013). Damage progression and defect sensitivity: an experimental study of representative wrinkles in tension. Compos B.

[29] Hsiao HM, Daniel IM (1996). Effect of fibre waviness on stiffness and strength reduction of unidirectional composites under compressive loading. Compos Sci Technol.

[30] Garnich MR, Karami G (2004). Finite element for stiffness and strength of wavy fibre composites. J Compos Mater.

[31] Karami G, Garnich MR (2005). Effective moduli and failure consideration for composites with periodic fibre waviness. Compos Struct.

[32] Cinar K, Ersoy N (2015). Effect of fibre wrinkling to the spring-in behaviour of L-shaped composite materials. Compos Part A.

[33] Potter K, Langer C, Hodgkiss B, Lamb S (2007). Sources of variability in uncured aerospace grade unidirectional carbon fibre epoxy preimpregnate. Compos A.

[34] Johnston A (1997) An integrated model of the development of process-induced deformation in autoclave processing of composite structures. Ph.D. thesis, The University of British Columbia

[35] Bogetti TA, Gillespie JW (1992). Process-induced stress and deformation in thick-section thermoset composite laminates. J Compos Mater.

[36] Bogetti TA, Gillipse JW (1989) Processing-induced stress and deformation in thick section thermosetting composite laminates. CCM report. University of Delaware, August, pp 89–21

[37] Ruiz E, Trochu F (2005). Numerical analysis of cure temperature and internal stresses in thin and thick RTM parts. Compos A.

[38] Wijskamp S (2005) Shape distortions in composites forming. PhD thesis, University of Twente, The Netherlands

[39] Baran I, Tutum CC, Hattel JH (2013). The effect of thermal contact resistance on the thermosetting pultrusion process. Compos B Eng.

[40] Baran I, Hattel JH, Tutum CC (2013). Thermo-chemical modelling strategies for the pultrusion process. Appl Compos Mater.

[41] Liu XL, Crouch IG, Lam YC (2000). Simulation of heat transfer and cure in pultrusion with a general-purpose finite element package. Compos Sci Technol.

[42] Baran I, Tutum CC, Hattel JH (2013). Optimization of the thermosetting pultrusion process by using hybrid and mixed integer genetic algorithms. Appl Compos Mater.

[43] Baran I, Tutum CC, Hattel JH (2013). Reliability estimation of the pultrusion process using the first- order reliability method (FORM). Appl Compos Mater.

[44] Ersoy N, Garstka T, Potter K, Wisnom MR, Porter D, Clegg M, Stringer G (2010). Development of the properties of a carbon fibre reinforced thermosetting composite through cure. Compos A.

[45] Akkerman R (2002). On the properties of quasi-isotropic laminates. Compos B Eng.

[46] Tsai SW, Hahn HT (1980). Introduction to composite materials.

[47] Goetschel DB, Radford DW (1997). Analytical development of through-thickness properties of composite laminates. J Adv Mater.

[48] Ersoy N, Tugutlu M (2010). Cure kinetics modelling and cure shrinkage behaviour of a thermosetting composite. Polym Eng Sci.

[49] Monti M, Puglia D, Natali M, Torre L, Kenny JM (2011). Effect of carbon nanofibers on the cure kinetics of unsaturated polyester resin: thermal and chemorheological modelling. Compos Sci Technol.

[50] Kosar V, Gomzi Z (2001). Thermal effects of cure reaction for an unsaturated polyester in cylindrical moulds. Chem Biochem Eng Q.

[51] Martin JL, Cadenato A, Salla JM (1997). Comparative studies on the non-isothermal DSC curing kinetics of an unsaturated polyester resin using free radicals and empirical models. Thermochim Acta.

[52] De la Caba K, Guerrero P, Mondragon I, Kenny JM (1998). Comparative study by and DSC FTIR techniques of an unsaturated polyester resin cured at different temperatures. Polym Int.

[53] Avella M, Martuscelli E, Mazzola M (1985). Kinetic study of the cure reaction of unsaturated polyester resins. J Therm Anal.

[54] Vilas JL, Laza JM, Garay MT, Rodriguez M, Leon LM (2001). Unsaturated polyester resins cure: kinetic, rheologic, and mechanical-dynamical analysis. I. Cure kinetics by DSC and TSR. J Appl Polym Sci.

[55] Kenny JM (1994). Determination of autocatalytic kinetic model parameters describing thermoset cure. J Appl Polym Sci.

[56] Kessler MR, White SR (2002). Cure kinetics of the ring-opening metathesis polymerization of dicyclopentadiene. J Poly Sci A Poly Chem.

[57] Baran I, Akkerman R, Hattel JH (2014). Material characterization of a polyester resin system for the pultrusion process. Compos B Eng.

[58] Johnston A, Vaziri R, Poursartip A (2001). A plane strain model for process-induced deformation of laminated composite structures. J Compos Mater.

[59] Khoun L, Hubert P (2010). Investigation of the dimensional stability of carbon epoxy cylinders manufactured by resin transfer moulding. Compos A.

[60] Chachad YR, Roux JA, Vaughan JG, Arafat E (1995). Three-dimensional characterization of pultruded fiberglass-epoxy composite materials. J Reinf Plast Comp.

[61] Valliappan M, Roux JA, Vaughan JG, Arafat ES (1996). Die and post-die temperature and cure in graphite-epoxy composites. Compos Part B Eng.

[62] Khoun L, Centea T, Hubert P (2010). Characterization methodology of thermoset resins for the processing of composite materials - case study: CYCOM 890RTM epoxy resin. J Compos Mater.

[63] Khoun L (2009) Process-induced stresses and deformations in woven composites manufactured by resin transfer moulding. Ph.D. thesis, McGill University, Montreal

[64] Nielsen MW (2012) Predictions of process induced shape distortions and residual stresses in large fibre reinforced composite laminates, Ph.D. thesis, Technical University of Denmark, Lyngby, Denmark

[65] De la Caba K, Guerrero P, Eceiza A, Mondragon I (1996). Kinetic and rheological studies of an unsaturated polyester cured with different catalyst amounts. Polymer.

[66] Svanberg JM, Altkvist C, Nyman T (2005). Prediction of shape distortions for a curved composite C-spar. J Reinf Plast Compos.

[67] Svanberg JM, Holmberg JA (2004). Prediction of shape distortions. Part II. Experimental validation and analysis of boundary conditions. Compos A.

[68] Ersoy N, Potter K, Wisnom MR, Clegg MJ (2005). An experimental method to study frictional processes during composite manufacturing. Compos A.

[69] Jonas A, Legras R (1993) Assessing the crystallinity of PEEK. In: Advanced thermoplastic composites-characterizing processing, Hanser, pp 84–109

[70] Avrami M (1939). Kinetics of phase change. I Gen Theory J Chem Phys.

[71] Avrami M (1940). Kinetics of phase change. II, transformation-time relations for random distribution of nuclei. J Chem Phys.

[72] Avrami M (1941). Kinetics of phase change. III, granulation, phase change and microstructure. J Chem Phys.

[73] Cebe P, Hong S-D (1986). Crystallization behavior of poly (ether–ether–ketone). Polymer.

[74] Velisaris CN, Seferis JC (1986). Crystallization kinetics of polyetheretherketone (PEEK) matrices. Polym Eng Sci.

[75] White SR, Hahn HT (1992). Process modelling of composite materials: residual stress development during cure. Part II. Experimental validation. J Compos Mater.

[76] Russell JD, Madhukar MS, Genidy MS, Lee AY (2000). A new method to reduce cure-induced stresses in thermoset polymer composites, part III: correlating stress history to viscosity, degree of cure, and cure shrinkage. J Compos Mater.

[77] Msallem YA, Jacquemin F, Boyard N, Poitou A, Delaunay D, Chatel S (2010). Material characterization and residual stresses simulation during the manufacturing process of epoxy matrix composites. Compos A.

[78] Prasatya P, McKenna GB, Simon SL (2001). A viscoelastic model for predicting isotropic residual stresses in thermosetting materials: effects of processing parameters. J Compos Mater.

[79] Madhukar MS, Genidy MS, Russell JD (2000). A new method to reduce cure-induced stresses in thermoset polymer composites, part I:test method. J Compos Mater.

[80] Dong C, Zhang C, Liang Z, Wang B (2004). Dimension variation prediction for composite with finite element analysis and regression modelling. Compos A.

[81] Hsiao KT, Gangireddy S (2008). Investigation on the spring-in phenomenon of carbon nanofibre-glass fibre/polyester composites manufactured with vacuum assisted resin transfer moulding. Compos A.

[82] Wisnom MR, Ersoy N, Potter KD (2007). Shear-lag analysis of the effect of thickness on spring-in of curved composites. J Compos Mater.

[83] Ersoy N, Garstka T, Potter K, Wisnom MR, Porter D, Stringer G (2010). Modelling of the spring-in phenomenon in curved parts made of a thermosetting composite. Compos A.

[84] Lawrence WE, Seferis JC, Gillespie JW (1992). Material response of a semicrystalline thermoplastic polymer and composite in relation to process cooling history. Polym Compos.

[85] Hubert P, Vaziri R, Poursartip A (1999). A two-dimensional flow model for the process simulation of complex shape composite laminates. Int J Numer Meth Eng.

[86] Min L, Yanxia L, Yizhuo G (2008). Numerical simulation flow and compaction during the consolidation of laminated composites. Wiley Intersci Soc Plast Eng.

[87] Dave R, Kardos JL, Dudukovic MP (1987). A model for resin flow during composite processing: part 1-general mathematical development. Polym Compos.

[88] Gutowski TG, Cai Z, Bauer S, Boucher D (1987). Consolidation experiments for laminate composites. J Compos Mater.

[89] Li M, Li Y, Zhang Z, Gu Y (2008). Numerical simulation flow and compaction during the consolidation of laminated composites. Polym Compos.

[90] Li M, Charles L, Tucker III (2002). Modelling and simulation of two-dimensional consolidation for thermoset matrix composites. Compos A.

[91] Dong C (2011). Model development for the formation of resin-rich zones in composites processing. Compos A.

[92] Svanberg JM (2002) Predictions of manufacturing induced shape distortions-high performance thermoset composites. PhD thesis, Lulea University of Technology

[93] Lamers EAD, Akkerman R, Wijskamp S (2003). Fibre orientation modelling for rubber press forming of thermoplastic laminates. Int J Form Process.

[94] Baran I (2014) Modelling the pultrusion process of off shore wind turbine blades, Ph.D. thesis, Technical University of Denmark, Lyngby, Denmark

[95] Kim YK (2004). Process-induced residual stress analysis by resin transfer molding. J Compos Mater.

[96] Kim YK, White SR (1996). Stress relaxation behavior of 3501–6 epoxy resin during cure. Polym Eng Sci.

[97] Kim YK, White SR (1997). Viscoelastic analysis of processing-induced residual stresses in thick composite laminates. Mech Compos Mater Struct.

[98] White SR, Kim YK (1998). Process-induced residual stress analysis of AS4/3501-6 composite material. Mech Compos Mater Struct Int J.

[99] Kim YK, White SR (1999). Cure-dependent viscoelastic residual stress analysis of filament-wound composite cylinders. Mech Compos Mater Struct.

[100] Ding A, Li S, Wang J, Zu L (2015). A three-dimensional thermo-viscoelastic analysis of process-induced residual stress in composite laminates. Compos Struct.

[101] Ding A, Li S, Sun J, Wang J, Zu L (2015) A thermo-viscoelastic model of process-induced residual stresses in composite structures with considering thermal dependence. Compos Struct. doi:10.1016/j.compstruct.2015.09.014

[102] Zobeiry N (2006) Viscoleastic constitutive models for evaluation of residual stresses in thermoset composites during cure. PhD thesis, The University of British Columbia

[103] Zocher MA (1995) A Thermoviscoelastic finite element formulation for the analysis of composites. PhD. thesis, Texas AM University

[104] Abouhamzeh M, Sinke J, Jansen K, Benedictus R (2015). Kinetic and thermo-viscoelastic characterisation of the epoxy adhesive in GLARE. Compos Struct.

[105] Zarrelli M, Partridge IK, D’Amore A (2006). Warpage induced in bi-material specimens: coefficient of thermal expansion chemical shrinkage and viscoelastic modulus evolution during cure. Compos A Appl Sci Manuf.

[106] Li H, Zhang B, Bai G (2015) A new method of characterizing material viscoelastic property by using vector fitting. Polym Compos. doi:10.1002/pc.23359

[107] O’Brien DJ, Mather PT, White SR (2001). Viscoelastic properties of an epoxy resin during cure. J Compos Mater.

[108] Cinar K, Oztrk UE, Ersoy N, Wisnom MR (2014). Modelling manufacturing deformations in corner sections made of composite materials. J Compos Mater.

[109] Cinar K (2014) Process modelling for distortions in manufacturing of fibre reinforced composite materials. Ph.D. thesis, Bogazici University

[110] Arafath ARA, Vaziri R, Poursartip A (2008). Closed-from solution for process-induced stresses and deformation of a composite part cured on a solid tool: Part I- flat geometries. Compos A.

[111] Arafath ARA, Vaziri R, Poursartip A (2009). Closed-from solution for process-induced stresses and deformation of a composite part cured on a solid tool: Part II- curved geometries. Compos A.

[112] Ferlund G, Rahman N, Courdji R, Bresslauer M, Poursartip A, Willden K, Nelson K (2002). Experimental and numerical study of the effect of cure cycle, tool surface, geometry, and lay-up on the dimensional fidelity of autoclave-processed composite parts. Compos A.

[113] Zhu Q, Geubelle PH, Li M, Tucker CL (2001). Dimensional accuracy of thermoset composites: simulation of process-induced residual stresses. J Compos Mater.

[114] Flanagan R (1997) The dimensional stability of composite laminates and structures. Ph.D. thesis, Queen’s University of Belfast

[115] Ozsoy OO, Ersoy N, Wisnom MR (2007) Numerical Investigation of tool-part interactions in composite manufacturing. Proceedings of ICCM-16

[116] Twigg G, Poursartip A, Ferlund G (2004). Tool-part Interaction in composite processing. Part II: numerical modelling. Compos A.

[117] Sun J, Gu Y, Li Y, Li M, Zhang Z (2012). Role of tool-part interaction in consolidation of L-shaped laminates during autoclave process. Appl Compos Mater.

[118] Hibbit, Karlson, Sorensen Inc. (2004) ABAQUS online documentation, version 6.5.1

[119] Twigg G, Poursartip A, Ferlund G (2003). An experimental method for quantifying tool-part shear interaction during composites processing. Compos Sci Technol.

[120] Twigg G, Poursartip A, Ferlund G (2004). Tool-part interaction in composite processing. Part I: experimental investigation and analytical model. Compos A.

[121] Potter KD, Campbell M, Langer C, Wisnom MR (2005). The generation of geometrical deformations due to tool/part interaction in the manufacture of composite components. Compos A.

[122] Kappel E, Stefaniak D, Sprowitz T, Hhne C (2011). A semi-analytical simulation strategy and its application to warpage of autoclave-processed CFRP parts. Compos A.

[123] Stefaniak D, Kappel E, Sprowitz T, Hhne C (2012). Experimental identification of process parameters inducing warpage of autoclave-processed CFRP parts. Compos A.

[124] Zeng X, Raghavan J (2010). Role of tool-part interaction in process-induced warpage of autoclave-manufactured composite structures. Compos A.

[125] Kaushik V, Raghavan J (2010). Experimental study of tool-part interaction during autoclave processing of thermoset polymer composite structures. Compos A.

[126] de Oliveria R, Lavanchy S, Chatton R, Costantini D, Michaud V, Salathe R, Manson JAE (2008). Experimental investigation of the mould thermal expansion on the development of internal stresses during carbon fibre composite processing. Compos A.

[127] Potter K, Campbell M, Wisnom MR (2003) Investigation of tool/part interaction effects in the manufacture of composite components. Proceedings of ICCM-14

[128] Kim YK, Daniel IM (2002). Cure cycle effect on composite structures manufactured by resin transfer moulding. J Compos Mater.

[129] Antonucci V, Cusano A, Giordano M, Nasser J, Nicolais L (2006). Cure- induced residual strain build-up in a thermoset resin. Compos A.

[130] Martin CJ, Seferis JC, Wilhelm MA (1996). Frictional resistance of thermoset prepregs and its influence on honeycomb composite processing. Compos A.

[131] ten Thije RHW, Akkerman R, Ubbink M, van der Meer L (2011). A lubrication approach to friction in thermoplastic composites forming processes. Compos A Appl Sci Manuf.

[132] Sachs U, Akkerman R, Fetfatsidis K, Vidal-Sall E, Schumacher J (2014). Characterization of the dynamic friction of woven fabrics: experimental methods and benchmark results. Compos A Appl Sci Manuf.

[133] Cornelissen B, Sachs U, Rietman B, Akkerman R (2014). Dry friction characterisation of carbon fibre tow and satin weave fabric for composite applications. Compos A Appl Sci Manuf.

[134] White SR, Hahn HT (1992). Process modelling of composite materials: residual stress development during cure. Part I. Model formulation. J Compos Mater.

[135] Loos AC, Springer GS (1983). Curing of epoxy matrix composites. J Compos Mater.

[136] Theriault RP, Osswald TA (1999). Processing induced residual stress in asymmetric laminate panles. Poly Compos.

[137] Wiersma HW, Peeters LJB, Akkerman R (1998). Prediction of springforward in continuous-fibre/polymer L-shaped parts. Compos A.

[138] Clifford S, Jansson N, Yu W, Michaud V, Manson JA (2006). Thermoviscoelastic anisotropic analysis of process induced residual stresses and dimensional stability in real polymer matrix composite components. Compos A.

[139] Tavakol B, Roozbehjavan P, Ahmed A, Das R, Joven R, Koushyar H, Rodriguez A, Minaie B (2013). Prediction of residual stresses and distortion in carbon fiber-epoxy composite parts due to curing process using finite element analysis. J Appl Polym Sci.

[140] Wucher B, Lani F, Pardoen T, Bailly C, Martiny P (2014). Tooling geometry optimization for compensation of cure-induced distortions of a curved carbon/epoxy C-spar. Compos A.

[141] Ferlund G, Osooly A, Poursartip A, Vaziri R, Courdji R, Nelson K, George P, Hendrickson L, Griffith J (2003). Finite element based prediction of process-induced deformation of autoclaved composite structures using 2D process analysis and 3D structural analysis. Compos Struct.

[142] Dong C (2009). Modeling the process-induced dimensional variations of general curved composite components and assemblies. Compos A.

[143] Alam MK, Anghelescu MS (2009). Analysis of deformation and residual stresses in composites processed on a carbon foam tooling. J Compos Mater.

[144] Guo ZS, Zhang J, Guo X, Hu H (2008) Theoretical and experimental characteristics on residual stresses of advanced polymer composites. The 15th international symposium on: smart structures and materials and nondestructive evaluation and health monitoring

[145] Jun L, XueFeng Y, YingHua L, ZhangZhi C, ZheJun K, XiaoCai H, Di D (2010). Thermo-viscoelastic analysis of the integrated T-shaped composite structures. Compos Sci Technol.

[146] Olivier PA, El Sawi I (2010). Designing curing conditions in order to analyse the influence of process-induced stresses upon some mechanical properties of carbon/epoxy laminates at constant Tg and degree of cure. Int J Mater Form.

[147] Tarsha-Kurdi KE, Olivier P (2002). Thermoviscoelastic analysis of residual curing stresses and the influence of autoclave pressure on these stresses in carbon/epoxy laminates. Compos Sci Technol.

[148] Huang X, Gillespie JW, Bogetti T (2000). Process induced stress for woven fabric thick section composite structures. Compos Struct.

[149] Golestanian H, El-Gizawy S (2001). Modeling of process induced residual stresses in resin transfer molded composites with woven fiber mats. J Compos Mater.

[150] Dong C, Zhang C, Liang Z, Wang B (2004). Assembly dimensional variation modelling and optimization for the resin transfer moulding process. Modell Simul Mater Sci Eng.

[151] Khoun L, de Oliveira R, Michaud V, Hubert P (2011). Investigation of process-induced strains development by fibre Bragg grating sensors in resin transfer moulded composites. Compos A.

[152] Baran I, Tutum CC, Nielsen MW, Hattel JH (2013). Process induced residual stresses and distortions in pultrusion. Compos B Eng.

[153] Baran I, Hattel JH, Akkerman R, Tutum CC (2015). Mechanical modelling of pultrusion process: 2D and 3D numerical approaches. Appl Compos Mater.

[154] Carlone P, Baran I, Hattel JH, Palazzo GS (2013) Computational approaches for modelling the multi-physics in pultrusion process. Adv Mech Eng 2013, Article ID 301875, 14 pages. doi:10.1155/2013/301875

[155] Baran I, Tutum CC, Hattel JH (2013). The internal stress evaluation of the pultruded blades for a Darrieus wind turbine. Key Eng Mater.

[156] Baran I, Tutum CC, Hattel JH, Akkerman R (2015). Pultrusion of a vertical axis wind turbine blade part-I: 3D thermo-chemical process simulation. Int J Mater Form.

[157] Baran I, Hattel JH, Tutum CC, Akkerman R (2015). Pultrusion of a vertical axis wind turbine blade part-II: combining the manufacturing process simulation with a subsequent loading scenari. Int J Mater Form.

[158] Baran I, Hattel JH, Akkerman R (2015). Investigation of process induced warpage for pultrusion of a rectangular hollow profile. Compos B Eng.

[159] Baran I, Akkerman R, Hattel JH (2014). Modelling the pultrusion process of an industrial L-shaped composite profile. Compos Struct.

[160] Zenkert D, Battley M (2009). Laminate and sandwich structures: foundations of fibre composites.

[161] Nielsen MW, Schmidt JW, Hattel JH, Andersen TL, Markussen CM (2013). In situ measurement using FBGs of process induced strains during curing of thick glass/epoxy laminate plate: experimental results and numerical modelling. Wind Energy.

[162] Velisaris CN, Seferis JC (1988). Heat transfer effects on the processing-structure relationships of polyetheretherketone (PEEK) based composites. Polym Eng Sci.

[163] Hwang SJ, Tucker CL (1990). Heat transfer analysis of continuous fiber/thermoplastic matrix composites during manufacture. J Thermoplast Compos Mater.

[164] Sonmez FO, Hahn HT (1997). Modeling of heat transfer and crystallization in thermoplastic composite tape placement process. J Thermoplast Compos Mater.

[165] Grove SM (1988). Thermal modelling of tape laying with continuous carbon-fibre-reinforced thermoplastic. Composites.

[166] Andersen BJ, Colton JS (1990). Automation of thermoplastic composite processing. J Compos Mater.

[167] Beyeler EP, Guceri SI (1988). Experimental investigation of laser assisted thermoplastic tape consolidation. J Compos Mater.

[168] Nejhad MN, Cope RD, Guceri SI (1988). Thermal analysis of in-situ thermoplastic-matrix composite filament winding. J Heat Trans.

[169] Lee WI, Springer GS (1987). A model of the manufacturing process of thermoplastic matrix composites. J Compos Mater.

[170] Sonmez FO, Hahn HT (1997). Analysis of the on-line consolidation process in thermoplastic composite tape placement. J Compos Mater.

[171] Pitchumani R, Gillespie JW, Lamontia MA (1997). Design and optimization of a thermoplastic tow-placement process with in-situ consolidation. J Compos Mater.

[172] Tierney J, Gillespie JW (2006). Modeling of in situ strength development for the thermoplastic composite tow placement process. J Compos Mater.

[173] Chapman TJ, Gillespie JW, Pipes RB, Manson J-AE, Seferis JC (1990). Prediction of process induced residual stresses in thermoplastic composites. J Compos Mater.

[174] Ersoy N, Vardar O (2000). Measurement of residual stresses in layered composites by compliance method. J Compos Mater.

[175] Li MC, Wu JJ, Loos AC (1997). Plane-strain finite element model for process-induced residual stresses in a graphite/PEEK composite. J Compos Mater.

[176] Mantell SC, Springer GS (1992). Manufacturing process models for thermoplastic composites. J Compos Mater.

[177] Tierney J, Gillespie JW (1998) Control of warpage and residual stresses during the automated tow placement, SOC PLAST ENGINEERS, 56th annual technical conference of the Society-of-Plastics-Engineers-Plastics on My Mind (ANTEC 98), ATLANTA, GA, Apr 26–30

[178] Sonmez FO, Hahn HT, Akbulut M (2002). Analysis of process-induced residual stresses in tape placement. J Thermoplast Compos Mater.

[179] Trende A, Astrom BT, Nilsson G (2000). Modelling of residual stresses in compression moulded glass-mat reinforced thermoplastics. Compos A Appl Sci Manuf.

[180] Kim BS, Bernet N, Sunderland P, Manson JA (2002). Numerical analysis of the dimensional stability of thermoplastic composites using a thermoviscoelastic approach. J Compos Mater.

[181] Brauner C, Peters C, Brandwein F (2014). Analysis of process-induced deformations in thermoplastic composite materials. J Compos Mater.

[182] Lynam C, Milani AS, Trudel-Boucher D (2014). Predicting dimensional distortions in roll forming of comingled polypropylene/glass fiber thermoplastic composites: On the effect of matrix viscoelasticity. J Compos Mater.

[183] Akkerman R, Lamers EAD, Wijskamp S (2006). An integral process model for high precision composite forming. Eur J Comput Mech.

[184] Haanappel SP, ten Thije RHW, Sachs U, Rietman B, Akkerman R (2014). Formability analyses of uni-directional and textile reinforced thermoplastics. Compos A.

[185] AniForm virtual forming. http://www.aniform.com

[186] Dodwell TJ, Butler R, Hunt GW (2014). Out-of-plane ply wrinkling defect during consolidation over an external radius. Compos Sci Technol.

